# Platelet activation, aspirin, and cancer: From basic science to clinical trials

**DOI:** 10.1016/j.pharmr.2025.100109

**Published:** 2025-12-02

**Authors:** Carlo Patrono, John Burn, Paola Patrignani, Ruth E. Langley

**Affiliations:** 1 Department of Cardiovascular and Pulmonary Sciences, Catholic University School of Medicine, Rome, Italy; 2 Translational and Clinical Research Institute, Newcastle University, Centre for Life, Newcastle upon Tyne, United Kingdom; 3 Department of Neuroscience, Imaging and Clinical Sciences and Center for Advanced Studies and Technology (CAST), “G. d’Annunzio” University of Chieti-Pescara, Chieti, Italy; 4 MRC Clinical Trials Unit at UCL, University College London, London, United Kingdom

## Abstract

There is extensive evidence that aspirin prevents cancer, but the mechanism of action is uncertain. Once-daily low-dose aspirin (75–100 mg) completely and permanently inactivates the cyclooxygenase (COX) activity of prostaglandin G/H synthase-1 (COX-1) in platelets, suppressing thromboxane (TX)A_2_-dependent platelet activation. In this article, we review the mechanistic links between platelet activation, inflammation, cancer development, and progression and summarize recent clinical trial results and associated biomarker studies. We hypothesize that persistently enhanced platelet activation has 2 distinct tumorigenic consequences mediated by the release of TXA_2_: (1) at sites of gastrointestinal mucosal lesions, it promotes a local inflammatory response with COX-2 induction and enhanced prostaglandin E_2_ biosynthesis, contributing to early events in carcinogenesis; (2) it inhibits T-cell immunity to cancer by the activation of TXA_2_ receptors in lymphocytes, promoting cancer progression and metastasis dissemination. Supporting these hypotheses, abnormal and persistent platelet activation has been demonstrated in patients recently diagnosed with cancer and in those with adenomatous colonic polyps. To date, most clinical trials evaluating aspirin have focused on either primary cancer prevention, metastasis prevention (adjuvant treatment), or cardiovascular prevention. For an individual, benefits may accrue from one (or all) of these areas, and they collectively need to be balanced against bleeding risk. Collating large clinical datasets for meta-analysis alongside mechanistic studies will inform the interpretation of clinical trials, with the aim of identifying individuals most likely to benefit from aspirin.

## I Introduction

There is considerable preclinical, epidemiological, and randomized evidence that aspirin prevents cancer, but the mechanism of action is only partially characterized. It is well-established that a once daily regimen of low-dose aspirin (75–100 mg) completely and permanently inactivates the cyclooxygenase (COX) activity of prostaglandin (PG)G/H synthase-1 (colloquially referred to as COX-1) in platelets. This suppresses thromboxane (TX)A_2_–dependent platelet activation,^1^ though the wider patho-physiological and clinical consequences for cancer development and metastasis are less established. High levels of platelet activation are associated with both cardiovascular and cancer mortality during long-term follow-up.^2^ We have hypothesized and provided experimental evidence that persistently enhanced platelet activation has 2 distinct tumorigenic consequences mediated by the release of TXA_2_: (1) at sites of gastrointestinal (GI) mucosal lesions, it promotes a local inflammatory response with COX-2 induction and enhanced PGE_2_ biosynthesis, contributing to early events in carcinogenesis,^3^ and (2) it suppresses T-cell immunity to cancer by the activation of TXA_2_ receptors (TPs) in lymphocytes, promoting cancer progression and metastasis dissemination.^4^ The aim of this article is to review and discuss the basic and clinical evidence supporting these hypotheses. The history and use of antiplatelet strategies to combat or manage cancer and metastasis have been reviewed previously.^5^

Specifically, we will: (1) review the relationship between platelet activation, inflammation, and T-cell immunity to provide a mechanistic understanding of the anticancer effects of aspirin and, possibly, other antiplatelet agents, such as P2Y_12_ blockers (eg, clopidogrel), emphasizing what we know so far and indicating the knowledge gaps in the platelet hypothesis; (2) discuss how measurement of TXA_2_-dependent platelet activation may represent a biomarker of cancer risk, with potential to guide the use of anti-platelet drugs for cancer prevention; (3) review recently completed as well as ongoing clinical trials of aspirin for the primary and secondary prevention of cancer; (4) offer a perspective on what we would like to know regarding further mechanistic insights into platelet mediators contributing to cancer development and progression, the specificity of platelet target(s) for effective antiplatelet intervention, and the patient groups most likely to benefit from antiplatelet therapy.

### II Prostanoid biosynthesis and inhibition in health and disease

**Fig. 1:**
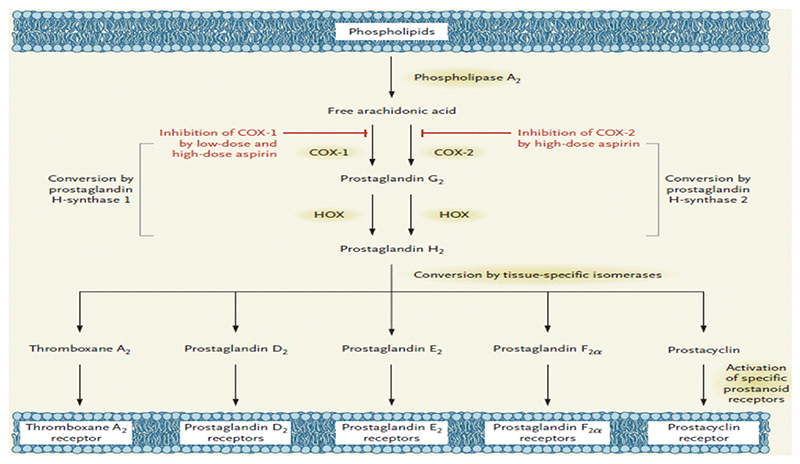
Prostanoid biosynthesis and inhibition. AA, a 20-carbon fatty acid containing 4 double bonds, is liberated from the sn2 position of membrane phospholipids by several forms of phospholipase A_2_, which are activated by diverse stimuli. AA is converted by cytosolic PGH synthases, which have both COX and hydroperoxidase (HOX) activity, to the unstable intermediates PGG_2_ and PGH_2_, respectively. The synthases are colloquially termed “cyclooxygenases” and exist in 2 forms, COX-1 and COX-2. Low-dose aspirin selectively inhibits COX-1, whereas high-dose aspirin and other traditional NSAIDs inhibit both COX-1 and COX-2. PGH_2_ is converted by tissue-specific isomerases to multiple prostanoids. These bioactive lipids activate specific cell-membrane receptors of the superfamily of G protein-coupled receptors, such as the TPs, the DPs, the EPs, the PGF_2*α*_ receptors, and the PGI_2_ receptor (IP). Reproduced from Patrono et al^1^ with permission from the Massachusetts Medical Society.

Prostanoids are produced by the isozymes COX-1 and COX-2 (Fig. 1), which are homodimers consisting of 576 and 581 amino acids, respectively. Each subunit contains COX and peroxidase active sites, which catalyze the conversion of free arachidonic acid (AA) to PGG_2_ and PGG_2_ to PGH_2_.^6^ AA is released from membrane phospholipids by cytosolic phospholipase A_2_ upon cellular activation. PGH_2_ is then converted into various prostanoids (prostacyclin [PGI_2_], PGE_2_, PGF_2*α*_, PGD_2_, and TXA_2_).^6^ The synthesis of PGE_2_ involves 3 synthases: cytosolic PGE-synthase and 2 membrane-bound synthases, microsomal PGE-synthase-1 and -2.^7^ While cytosolic PGE-synthase and mPGES-2 are constitutive, mPGES-1 is inducible, and its expression, along with COX-2, increases PGE_2_ production during inflammation and cancer initiation and progression.^7^ The synthesis of PGD_2_ is regulated by 2 enzymes: lipocalin-PGD-synthase and hematopoietic-PGD-synthase, which differ in their biochemical characteristics and functions.^8^ TXA_2_ and PGI_2_ are synthesized by TX-synthase and PGI-synthase, respectively.^9,10^

Prostanoids act by binding to and activating heptahelical transmembrane receptors, commonly known as G protein-coupled receptors, ie, the E prostanoid receptor (EP)1, EP2, EP3, and EP4 subtypes of the PGE_2_ receptor; the PGD_2_ receptors (DP1 and DP2, also known as CRTH-2); the PGF2*α* receptor (FP); the PGI2 receptor (IP); and the TP (Fig. 1). They transduce specific signals resulting from receptor activation and are distributed in a tissue-specific manner, which explains the different functional responses to prostanoid generation.^11^

**Fig. 2:**
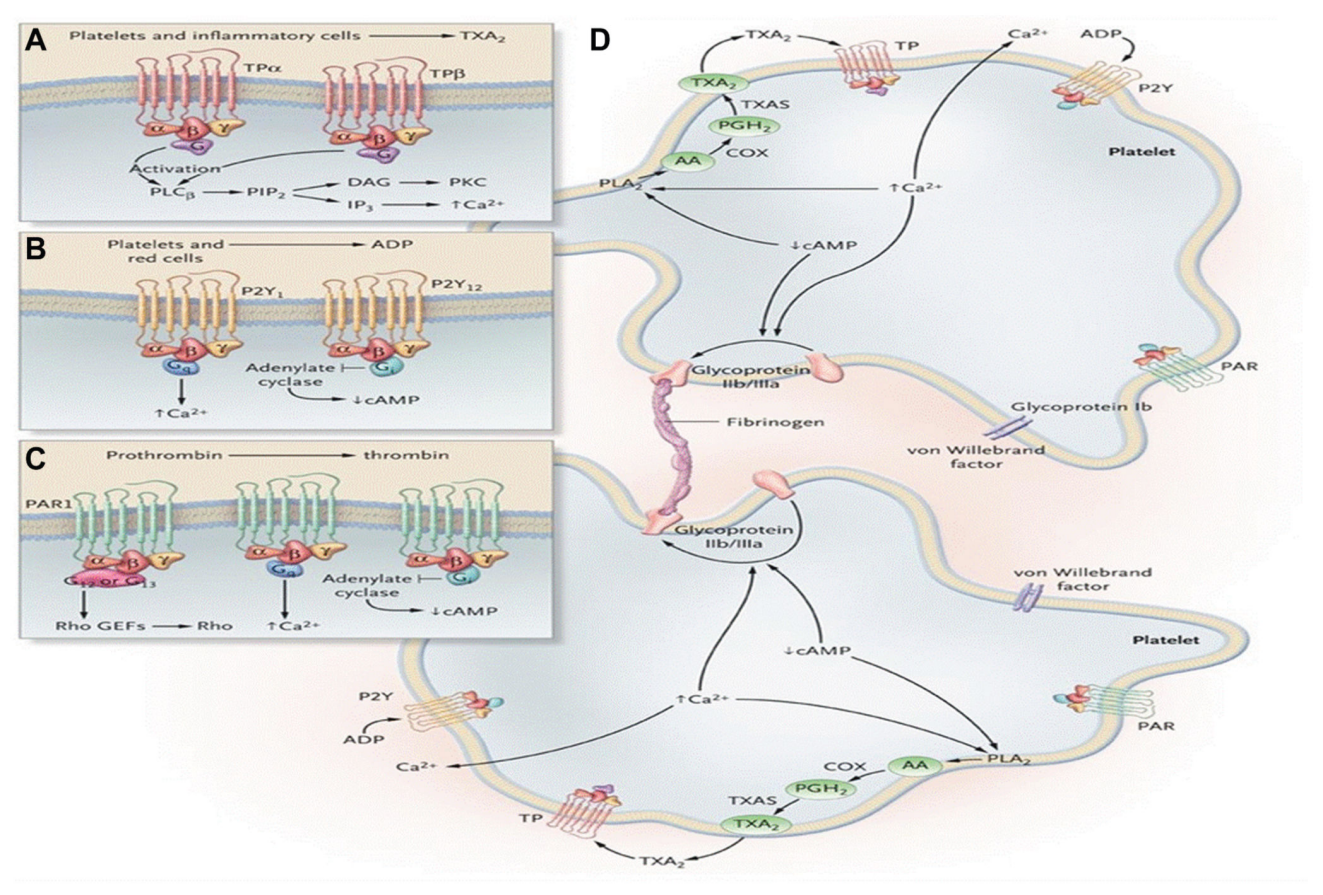
Agonists, receptors, and effector systems in platelet activation. The activation of platelets is induced by the interaction of several agonists with receptors expressed on the platelet membrane. (A–C) Outside-in signaling mediated by TXA_2_, ADP, and thrombin. (A) TXA_2_ is synthesized by activated platelets from AA through the COX pathway. Once formed, TXA_2_ can diffuse across the membrane and activate other platelets. In platelets, there are 2 splice variants of the TP: TP*α* and TP*ß*, which differ in their cytoplasmic tail. TP*α* and TP*ß* couple to the proteins Gq and G12 or G13, all of which activate phospholipase C (PLC). (B) ADP is released from platelets and red blood cells. Platelets express at least 2 ADP receptors, P2Y_1_ and P2Y_12_, which couple to Gq and Gi, respectively. The activation of P2Y_12_ inhibits adenylate cyclase, causing a decrease in cAMP levels, and the activation of P2Y_1_ causes an increase in intracellular Ca^2+^ levels. The P2Y_12_ receptor is the major receptor able to amplify and sustain platelet activation in response to ADP. (C) Thrombin is rapidly generated at sites of vascular injury from circulating prothrombin and, besides mediating fibrin generation, represents the most potent platelet activator. Platelet responses to thrombin are largely mediated through G protein-linked protease-activated receptors (PARs), which are activated after thrombin-mediated cleavage of their N-terminal exodomain. Human platelets express PAR1 and PAR4. PAR1 couples to members of the G12/13, Gq, and Gi protein families. (D) Inside-out signaling. The effects of agonists, mediated by decreases in cAMP levels and increases in intracellular Ca^2+^ levels, lead to platelet aggregation through changes in the ligand-binding properties of GPIIb/IIIa (*α*IIb*ß*3), which acquires the ability to bind soluble adhesive proteins such as fibrinogen and von Willebrand factor. The release of ADP and TXA_2_ induces further platelet activation and aggregation. PLA_2_, phospholipase A_2_; TXAS, thromboxane synthase. Reproduced from Davì and Patrono^15^ with permission from the Massachusetts Medical Society.

The TP is produced from a single gene with 2 splice variants: TP*α* and TP*ß*.^12^ TXA_2_ is one of the main mediators involved in the second phase of platelet activation during hemostasis and thrombosis, mainly via activation of the TP, which couples to Gq and G12/G13^13,14^ (Fig. 2).^15^ COX-1–dependent generation of TXA_2_ initiates an amplification loop that activates adjacent platelets, inducing further TXA_2_ formation. Similar to the inhibition of TXA_2_ biosynthesis by aspirin in humans, TP-deficient mice have platelets that are unable to form stable thrombi and show prolonged bleeding times.^16^ Besides involvement in primary hemostasis and thrombosis, human platelets also play an important physiologic role in tissue repair and regeneration. They are activated in response to injury, releasing a vast array of protein and lipid mediators that regulate fundamental mechanisms involved in the healing process, including cellular migration, proliferation, and angiogenesis.^17^

EP1, EP2, EP3, and EP4 have distinct pharmacologic signatures and intracellular signal transduction.^18^ Stimulation of EP1 and EP3 increases free intracellular Ca^2+^ levels. In contrast, stimulation of EP2 and EP4 increases intracellular cAMP levels through the activation of Gs protein, resulting in a decrease in intracellular Ca^2+^ levels.^18^

COX-1 and COX-2 have similar catalytic activities and produce the same products, but they serve different biological functions due to differences in gene expression, protein stability, and interactions with hydroperoxides and AA.^19^ COX-1 maintains a baseline level of prostanoid biosynthesis and enables a rapid increase in production when the availability of free AA levels is enhanced in response to cell-membrane perturbation. For instance, constitutive COX-1 expression in the GI mucosa supports the local synthesis of PGE_2_ and PGI_2_ for homeostatic cytoprotection. This protective mechanism operates by stimulating the synthesis and release of mucus and bicarbonate, inhibiting gastric acid secretion, enhancing blood flow to the mucosa, and promoting the proliferation of epithelial cells.^19^

In contrast, COX-2 is both a constitutive and an inducible enzyme. It enhances and sustains prostanoid production, particularly in response to inflammatory cytokines and growth factors, thereby forming an amplification loop that propagates prostanoid release from its focal origin to surrounding cells.^20,21^ The physiological induction of endothelial cell COX-2 by laminar shear stress is responsible for the biosynthesis and release of PGI_2_, a potent inhibitor of platelet activation that importantly contributes to endothelial thromboresistance.^22^

**Fig. 3:**
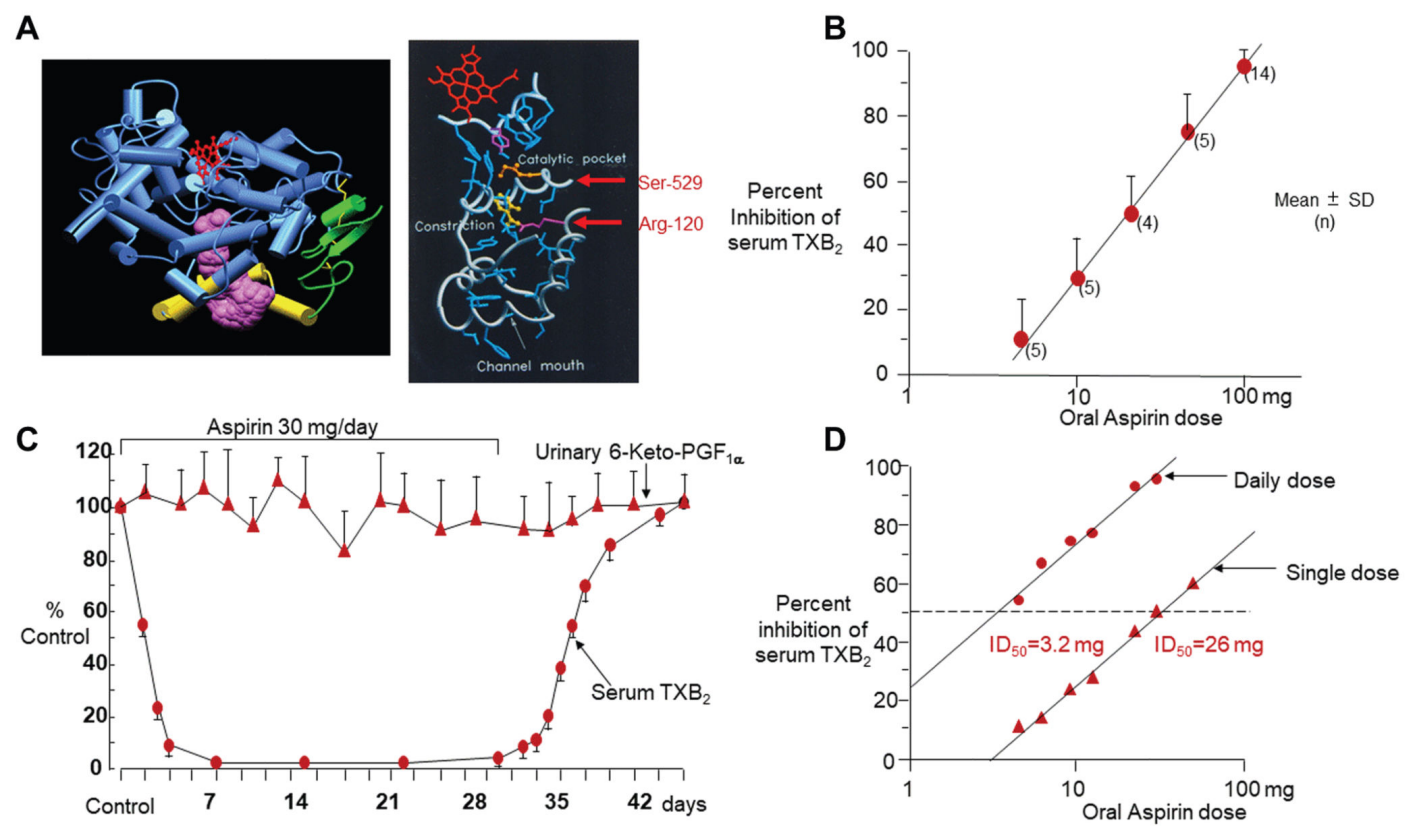
Aspirin antiplatelet pharmacodynamics in healthy subjects. (A) Tridimensional model of human PGG/H synthase-1. Acetylation of Ser-529 by aspirin permanently blocks the COX-1 channel near the catalytic pocket. (B) Inhibition of platelet TXB_2_ production by oral aspirin in healthy subjects. TXB_2_ production during whole-blood clotting was measured before and 24 hours after a single aspirin ingestion. The results are expressed as percent inhibition, each subject serving as their own control. Mean values ± 1 SD are plotted. Numbers in parentheses indicate the number of subjects for each dose of aspirin. (C) Long-term effects of low-dose (0.45 mg/kg per day) aspirin on platelet TXB_2_ and renal PGI_2_ synthesis. Serum TXB_2_ concentrations and urinary excretion of 6-keto-PGF_I*α*_ were measured in 3 healthy subjects before, during, and after aspirin therapy. Mean values ± SEM are plotted. (D) Dose dependence of the inhibition of platelet TXB_2_ production by aspirin. Serum TXB_2_ was measured before and after single (▲) or daily (●) dosing with aspirin in 4 healthy subjects. Individual data are expressed as percent inhibition, with each subject serving as their own control. Daily dosing values represent measurements obtained at steady-state inhibition. ID50, 50% inhibitory dose. Reproduced from Patrono^23^ under the terms of the Creative Commons Attribution-NonCommercial License.

The biosynthesis of prostanoids is inhibited by nonsteroidal anti-inflammatory drugs (NSAIDs). Low-dose aspirin provides a paradigm for COX isozyme-selective and cell-specific inhibition owing to its short half-life and unique ability to irreversibly inactivate COX-1 in platelets^1^ (Fig. 3).^23^ Other NSAIDs (eg, ibuprofen and diclofenac) lack these pharmacokinetic and pharmacodynamic features and do not achieve the same degree of persistent platelet COX-1 inhibition as with low-dose aspirin.^19^ The coxibs, such as rofecoxib and celecoxib, were developed to mimic the COX-2–dependent effects of traditional NSAIDs while sparing COX-1 activity in the GI mucosa and platelets in order to improve GI safety.^24^

**Fig. 4:**
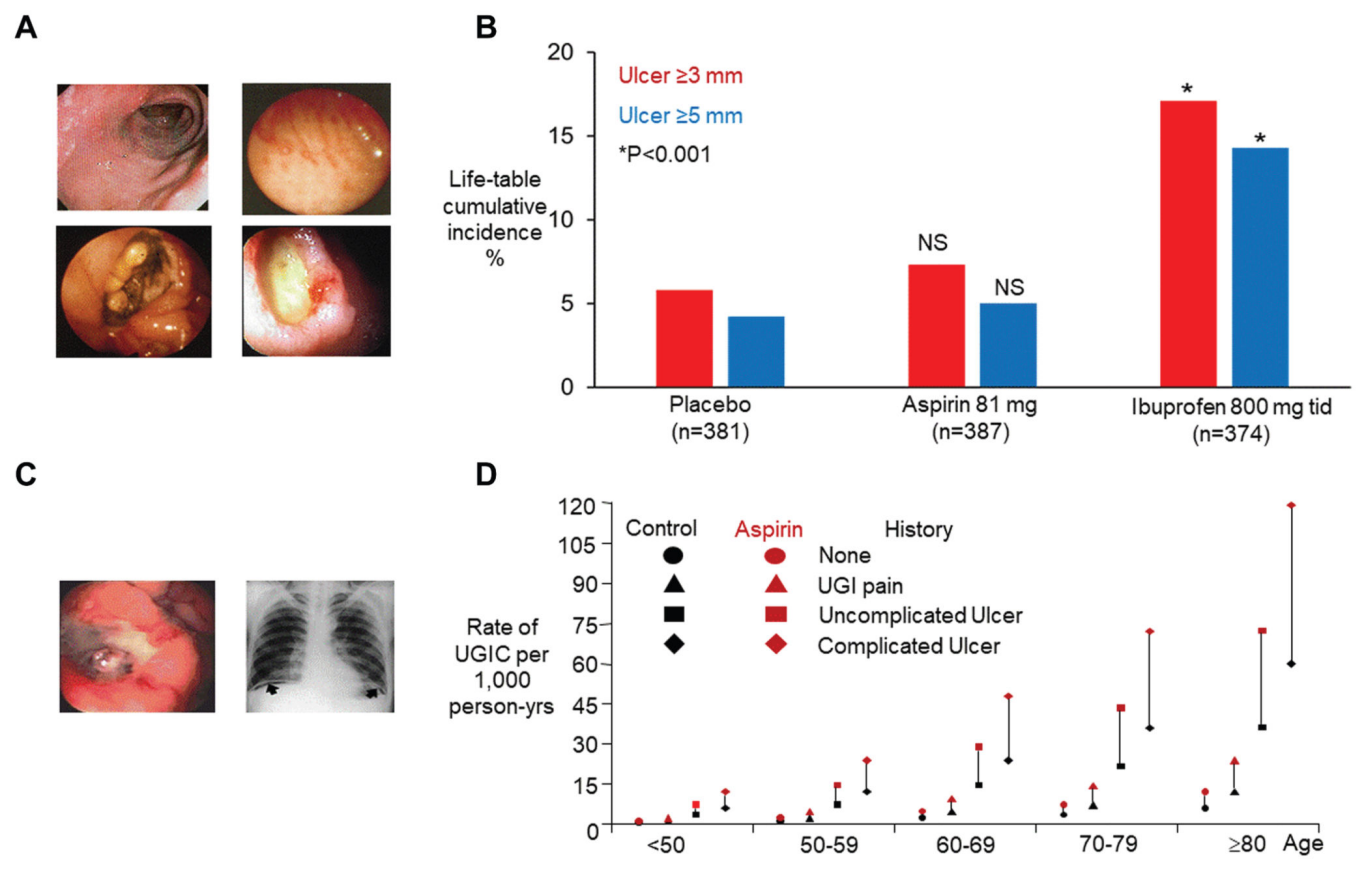
Low-dose aspirin does not cause new gastroduodenal ulcers but enhances the risk of bleeding from pre-existing lesions. (A) Gastroduodenal erosions and ulcers induced by NSAIDs. (B) Twelve-week cumulative incidences of gastroduodenal ulcers in osteoarthritis patients treated with low-dose aspirin, placebo, or ibuprofen. The figure was drawn with data from Laine et al.^26^ (C) GI complications induced by NSAIDs. (D) Estimated rates of upper GI (UGI) complications in men, according to age and the presence or absence of a history of such complications and regular treatment with low-dose aspirin. The vertical lines connecting each pair of black and red symbols depict the absolute excess of complications related to aspirin therapy. NS, not significant. Reproduced from Patrono^23^ under the terms of the Creative Commons Attribution-NonCommercial License. The asterisk corresponds to *P* < .01, as decribed in the upper left of panel B. UGIC, upper GI complications.

Permanent inactivation of platelet COX-1 by aspirin leads to the prevention of atherothrombosis but enhances the risk of bleeding, particularly GI bleeding. At least 2 distinct COX-1–dependent mechanisms may contribute to the increased risk of upper GI bleeding associated with aspirin exposure: inhibition of TXA_2_-mediated platelet function and impairment of PGE_2_-mediated cytoprotection of the GI mucosa.^25^ Whereas the former effect is dose-independent, for daily doses higher than 30 mg (Fig. 3), the latter effect is dose-dependent up to analgesic/anti-inflammatory dosing regimens (325–650 mg, 3–4 times daily). Inhibition of platelet function in primary hemostasis is largely responsible for the 2-fold increase in the risk of upper GI bleeding from preexisting mucosal lesions associated with daily doses of aspirin in the range of 75–100 mg. This is supported by a similar relative risk associated with other antiplatelet agents, such as clopidogrel, that do not inhibit COX activity.^23^ This interpretation is also supported by the results of a large, placebo-controlled, randomized trial of repeated upper GI endoscopy in elderly osteoarthritic patients.^26^ The 12-week cumulative incidence of gastro-duodenal ulcers of 7% associated with low-dose (81 mg daily) aspirin was not significantly higher than the placebo incidence of 6% (Fig. 4).^26^ The major risk factors for upper GI bleeding are older age and a previous history of GI disorders (Fig. 4).^1^

Inhibition of COX-1–dependent cytoprotection amplifies bleeding risk by causing new mucosal lesions and is associated with a relative risk of upper GI bleeding of about 4 at higher analgesic or anti-inflammatory doses of aspirin (ie, 325–650 mg, 4–6 times daily) or with traditional NSAIDs.^27^ The chronic use of selective COX-2 inhibitors, such as the coxibs and diclofenac, is associated with a 2-fold increase in upper GI bleeding.^27^ This finding most likely reflects a role for COX-2 induction at sites of GI mucosal injury and its participation in tissue repair, possibly in concert with local platelet activation in response to injury.^27^

Moreover, most COX-2 inhibitors increase the risk of serious vascular events, largely due to a doubling of the risk of myocardial infarction,^27^ consistent with an important role of COX-2 in endothelial thromboresistance.^22^

### III Analytical methods for measuring in vivo platelet activation

**Fig. 5:**
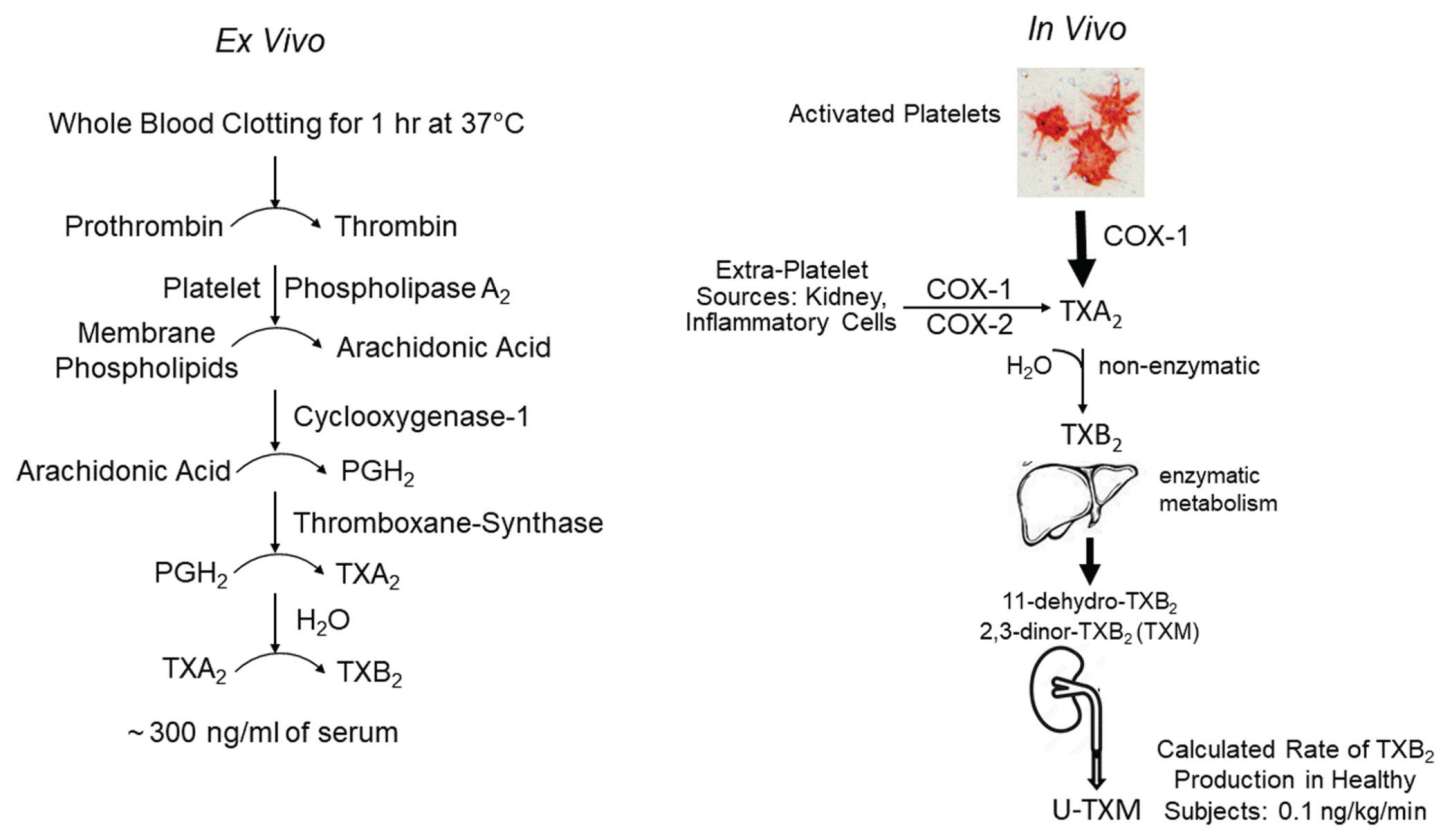
Assessment of platelet TXA_2_ biosynthesis ex vivo and in vivo. The left panel depicts the chain of enzymatic reactions triggered by thrombin generation during whole-blood clotting, leading to the formation of very large amounts of TXB_2_ (the stable hydration product of TXA_2_), which can be easily measured in serum as a sensitive and specific index of platelet COX-1 activity. The right panel depicts the metabolic fate of TXA_2_ in vivo and the calculated rate of its production in healthy subjects based on TXB_2_ infusions and measurement of its major urinary metabolites, 11-dehydro-TXB_2_ and 2,3-dinor-TXB_2_. The latter represents a noninvasive index of platelet activation in vivo.

Traditionally, platelet function has been investigated through measurements of platelet aggregation with the Born aggregometer^28^ and, more recently, with bedside devices based on the same physical principle.^29^ The ex vivo measurement of platelet responses to various agonists (eg, ADP or AA) provides an index of the functional capacity of platelets to react to exogenously added stimuli in a closed, artificial environment and of their pharmacologic inhibition by antiplatelet drugs.^29^ However, such measurements by no means reflect the true extent of platelet activation and inhibition in vivo. The maximum capacity of blood platelets to synthesize TXA_2_ in vitro during whole-blood clotting^30^ is at least 3 orders of magnitude higher than the calculated basal rate of TXA_2_ biosynthesis in vivo in healthy subjects (Fig. 5).^31^ Only a fraction of this biosynthetic capacity appears to contribute to pathophysiologic TXA_2_-dependent platelet activation, as reflected by the urinary excretion of TX metabolites (TXMs).^15^ On the other hand, the measurement of TXB_2_ and other platelet-derived products in plasma is hampered by the variable artifactual contribution of platelet activation during and after blood sampling.^32^ Immunoreactive TXB_2_ is undetectable in healthy human plasma when care is taken to avoid artifacts induced by sampling and when a high-sensitivity assay method is combined with a highly specific anti-body.^32^ In fact, based on the calculated rate of entry of endogenous TXB_2_ into the peripheral circulation and its total plasma clearance, a maximal estimate of the plasma concentration of endogenous TXB_2_ of 2.0 pg/mL was obtained in healthy subjects.^31^

When TXA_2_ was infused into cynomolgus monkeys,^33^ or TXB_2_ was infused into healthy volunteers,^31,34^ 2 major enzymatic metabolites were identified in the urine, namely 2,3-dinor-TXB_2_ and 11-dehydro-TXB_2_, colloquially referred to as TXM. These metabolites are the products of hepatic *ß*-oxidation and 11-OH-dehydrogenation of the parent compound(s), respectively.^35^ The rate of urinary TXM (U-TXM) excretion reflects the rate of entry of TXA_2_/TXB_2_ into the circulation and provides a time-integrated index of in vivo TXA_2_ biosynthesis.^31,32^ This conclusion is based on the following findings: (1) there is a linear relationship between the quantities of infused TXB_2_ in the human circulation, up to approximately 50-fold the calculated rate of secretion of endogenous TXB_2_, and the amount of TXM excreted in excess of control values in both healthy subjects^31,36^ and patients with diabetes mellitus, who have 4-fold higher TXM excretion,^37^ (2) the fractional elimination of the 2 major metabolites is independent of the rate of TXB_2_ infusion and yields almost identical estimates of the rate of entry of endogenous TXB_2_ into the human systemic circulation, ie, 0.11–0.12 ng/kg per minute,^31,36^ and (3) experiments performed in cynomolgus monkeys show a similar fractional conversion of TXA_2_ and TXB_2_ into 2,3-dinor-TXB_2_ and 11-dehydro-TXB_2_, suggesting that chemically unstable TXA_2_ is hydrolyzed nonenzymatically to TXB_2_ prior to enzymatic degradation.^33^

Urinary prostanoid metabolites reflect total body production of the parent compounds and usually cannot indicate a specific cellular source. However, the finding that low-dose aspirin suppressed U-TXM excretion by approximately 80% in healthy subjects^35,38^ and persons with diabetes mellitus,^37^ and that TXM excretion recovered over the next 10 days upon aspirin withdrawal, is consistent with a primary platelet source of U-TXM.^37,38^ When the effect of low-dose aspirin (100 mg daily) on U-TXM was examined over 8 consecutive weeks in 47 healthy subjects, the intrasubject coefficient of variation in percent inhibition was 21% ± 11%, a degree of intraindividual variability quite comparable with that observed in standard ex vivo measurements of platelet aggregation inhibition.^38^

#### A Tumor-educated platelets

Platelets isolated from cancer patients often display distinct RNA and protein profiles, with no clear alterations in hemostatic activity. This phenotypically distinct population, termed “tumor-educated platelets,” has received considerable attention for its potential use as a liquid biopsy for early cancer detection, despite substantial uncertainty about the mechanism(s) underlying platelet education.^39^ Indeed, tumor-educated platelet-derived RNA profiles can be employed to differentiate early- and late-stage cancer patients from healthy controls across several tumor types, enabling the detection of up to 18 different cancer types (including colorectal cancer [CRC]) and the localization of the primary tumor.^40^ However, due to the nature of high false-positive test results in patients with noncancerous diseases, the current pan-cancer test is practically applicable only to asymptomatic individuals.^40^ Furthermore, a control population of age- and gender-matched patients with evidence of persistent platelet activation (eg, due to diabetes mellitus and/or obesity) would be required in order to further assess the specificity of the pan-cancer test.

### IV Drivers of enhanced platelet activation in metabolic disorders and cancer

TXA_2_-dependent platelet activation, as reflected by U-TXM excretion, is persistently enhanced in association with major cardiovascular risk factors that accelerate atherogenesis,^37,41–44^ in patients with early-stage solid cancers,^45^ and in “high-risk” colorectal polyp patients.^46^

An increased generation of reactive oxygen species can induce enhanced lipid peroxidation of cell-membrane phospholipids or circulating low-density lipoproteins, leading to increased generation of F2-isoprostanes, a family of PG isomers produced from AA by an aspirin-insensitive mechanism catalyzed by free radicals.^47,48^ F2-isoprostanes, such as 8-iso-PGF_2*α*_, can modulate the adhesive reactions and activation of platelets induced by low levels of other agonists.^49^ Several studies have described a consistent relationship between the rates of formation of F2-isoprostanes and TXA_2_-dependent platelet activation in obese women and in patients with type 2 diabetes mellitus, hypercho-lesterolemia, or homozygous homocystinuria (reviewed by Davì and Patrono^15^). These findings suggest that a low-grade inflammatory state associated with a variety of metabolic disorders may represent an important trigger of TXA_2_-dependent platelet activation that is mediated, at least in part, by enhanced lipid peroxidation.^15^

Persistently enhanced platelet activation appears to occur early in the natural history of glucose control impairment, as suggested by a longitudinal study comparing subjects with impaired glucose tolerance (IGT) and those with established type 2 diabetes.^50^ Baseline U-TXM excretion was similar in 48 subjects with IGT and in 118 with established diabetes. During a 36-month followup, U-TXM excretion remained stable over time in subjects with diabetes, whereas it increased time-dependently in those with IGT. Increased U-TXM excretion over time was observed in the subjects who progressed to diabetes, but not in those who did not progress to diabetes.^50^

A substudy of the ASCEND (A Study of Cardiovascular Events in Diabetes) trial (NCT00135226), which evaluated the use of aspirin in persons with diabetes mellitus, assessed whether the basal rate of U-TXM excretion is associated with a risk of future serious vascular events or revascularizations (SVE-Rs) during 6.6 years of follow-up.^51^ U-TXM was measured prior to randomization to low-dose aspirin or placebo in 5948 participants with type 1 or 2 diabetes and no previous cardiovascular disease. Higher U-TXM was associated with older age, female sex, current smoking, type 2 diabetes, greater body size, a urinary albumin/creatinine ratio ≥ 3 mg/mmol, and a higher estimated glomerular filtration rate.^51^ After adjusting for these variables, baseline U-TXM was marginally significantly associated with SVE-Rs (hazard ratio [HR] per 1 SD higher logTXM, 1.09; 95% CI, 1.00–1.18). The size of the observational association was consistent with the effect size of subsequent randomization to aspirin if its statistically significant effect on SVE-Rs (12% relative risk reduction) was mediated by suppression of platelet TXA_2_ biosynthesis.^51^

Interestingly, although U-TXM excretion was not significantly associated with any cancer (HR, 1.06; 95% CI, 0.98–1.14), it was marginally associated with GI cancer (HR, 1.16; 95% CI, 1.00–1.36).^51^ However, despite more than 7 years of aspirin treatment and follow-up in ASCEND, no evidence of a reduction in the incidence of GI cancer or cancer at any other site, even during the later years of follow-up, was observed. These analyses, however, had limited statistical power to detect the hypothesized effects, so follow-up is being continued through central registries.^52^

A prospective observational study of 3044 participants in the Framingham Heart Study, followed over a median of 11.9 years, showed that among those not using aspirin, when adjusted for known cardiovascular risk factors, baseline U-TXM was positively associated with cardiovascular death (HR, 2.82; 95% CI, 1.39–5.66; *P* < .004 between quartile [Q]4 and Q1–3), but this was based on only 45 cardiovascular deaths, with no information on nonfatal events.^2^ In contrast to the ASCEND results, this study also reported a significant association between baseline U-TXM and cancer death in those not on aspirin (based on 117 cancer deaths: HR, 2.02; 95% CI, 1.39–2.92; *P* = .0002) and in the whole population (HR, 1.63; 95% CI, 1.24–2.13; *P* = .0005),^2^ but the degree to which this association was adjusted for potential confounders is not clear.

In a substudy of the Add-Aspirin trial (ISRCTN74358648), which is evaluating the adjuvant therapeutic effect of aspirin after radical cancer therapy, involving 716 cancer patients (breast 260, colorectal 192, gastroesophageal 53, and prostate 211), baseline median U-TXM excretion was higher in patients with cancer than in healthy individuals.^45^ Higher TXM levels were associated with higher body mass index (BMI), higher levels of inflammatory markers, and in patients with colorectal and gastro-esophageal cancer compared with patients with breast cancer, independent of other baseline characteristics.^45^ Interestingly, in a prostate cancer case-control study (977 cases and 1022 controls), higher levels of U-TXM were associated with a higher-risk disease and poorer outcomes.^53^

Based on these findings, it is tempting to speculate that U-TXM may serve as a noninvasive biomarker of cancer development and progression and that its modulation may be a marker for anti-platelet cancer therapy. Continued follow-up of ASCEND participants on cancer outcomes will allow testing this hypothesis.

### V Interplay between platelet activation, inflammatory and immune responses, and cancer development

In the last decade, the role of platelets beyond hemostasis and thrombosis has become more evident. Platelets release various mediators, including prostanoids, growth factors, ADP, and extracellular vesicles, which can influence inflammatory and immune responses, even at distant sites.^54^ For example, in a mouse model of corneal epithelial abrasion, platelet extravasation correlated with red blood cell extravasation and the disruption of microvascular integrity, and was dependent on *ß*_2_-integrin, CD18, neutrophils, and mast cell degranulation.^55^ During allergic inflammation, platelets are key to the pulmonary recruitment of eosinophils and lymphocytes.^56–58^ In hypertensive mice lacking the PGI_2_ receptor, increased TXA_2_ production was associated with cardiac fibrosis and platelet extravasation in the heart.^59^

In the setting of intestinal inflammation, such as colitis in mice or inflammatory bowel disease in humans, platelets have been shown to migrate from the bloodstream into the interstitial tissue of the colon.^60,61^ Coculturing human platelets with intestinal myofibroblasts activates platelets, leading to increased production of TXA_2_ through COX-1 activity. TXA_2_ influences myofibroblasts to adopt a polarized phenotype, enhancing their proliferation and migratory properties.^62^ These changes induced by platelets are associated with reduced *α*-smooth muscle actin and increased expression of COX-2, vimentin, fibronectin, and Ras homolog family member A, and are prevented by selective inhibition of platelet COX-1 or TP antagonism, suggesting a central role of TP signaling in this phenomenon.^3^ TXA_2_-dependent induction of COX-2 leads to enhanced PGE_2_ production by fibroblasts,^63^ and PGE_2_ activates tumor epithelial cells, leading to proliferation, survival, migration, invasion, and epigenetic alterations.^64^ Additionally, PGE_2_ facilitates tumor growth by inhibiting immune surveillance and promoting angiogenesis.^65^ Elevated levels of PGE_2_ in the tumor microenvironment may also inhibit apoptosis in epithelial cells,^66^ potentially promoting the accumulation of mutations.

**Fig. 6:**
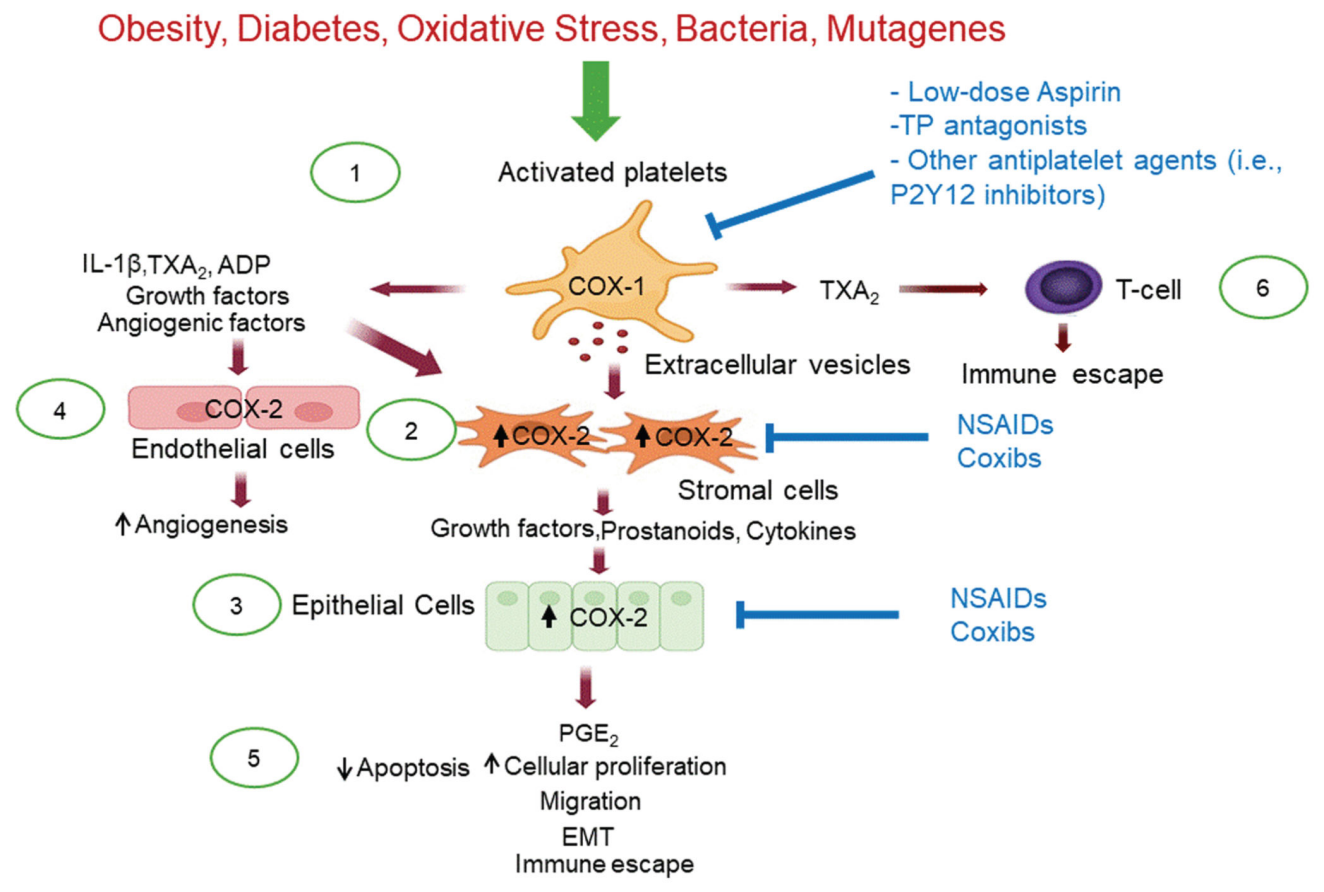
Platelet activation promotes the early stages of colorectal tumorigenesis. (1) Activated platelets release many mediators, including prostanoids (such as TXA_2_), ADP, cytokines (such as interleukin [IL]-1*ß*), growth and angiogenic factors, and extracellular vesicles containing genetic material (such as mRNAs and microRNAs) that contribute to (2) COX-2 induction in stromal cells (including macrophages and fibroblasts). (3) Crosstalk with epithelial cells leads to COX-2 induction. (4) Additionally, COX-2 is overexpressed in endothelial cells, leading to angiogenesis. (5) These events lead to the release of PGE_2_, which mediates inhibition of apoptosis, an increase in proliferation and migration, and induction of epithelial-mesenchymal transition (EMT). (6) TXA_2_ causes immunosuppression by inhibiting T-cell function. The inhibition of platelet COX-1 by low-dose aspirin and possibly other antiplatelet agents prevents the downstream signaling pathways involved in the early events of tumorigenesis, indirectly inhibits COX-2 induction, and reactivates the immune response. In contrast, NSAIDs and coxibs have an antitumor effect by directly inhibiting COX-2 activity and prostanoid generation in stromal, epithelial, and endothelial cells. Reproduced from Contursi et al^54^ under the terms of the Creative Commons CC BY license.

These lines of experimental evidence highlight the potential role of platelets in establishing an inflammatory microenvironment in the intestine during the initial stages of tumorigenesis through TXA_2_-mediated upregulation of COX-2 and increased PGE_2_ production (Fig. 6). They also suggest that inhibiting platelet activation with antiplatelet agents may disrupt this cascade, influencing the early events of intestinal tumorigenesis.^19^ Additionally, once cancer develops, many tumors overexpress COX-2, providing an additional therapeutic opportunity to achieve similar effects using either coxibs or traditional NSAIDs^19^ (Fig. 6).

**Fig. 7:**
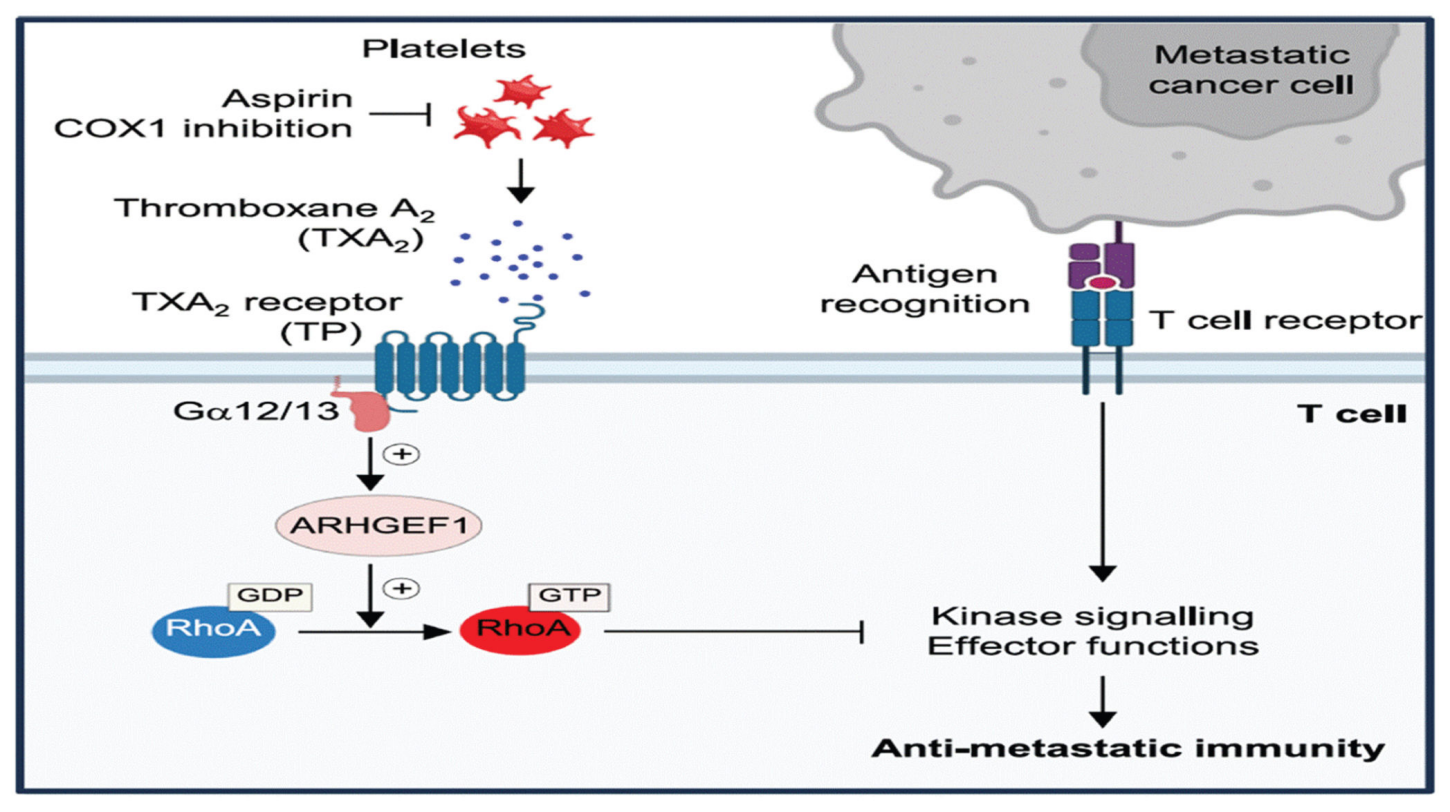
Aspirin promotes immunity to cancer metastasis by releasing T cells from TX-mediated suppression. Activated platelets synthesize and release TXA_2_, which binds to its receptor (TP) on T cells. This binding activates ARHGEF1, a guanine exchange factor that converts inactive GDP-bound Ras homolog family member A (RhoA) into its active GTP-bound form. The activation of RhoA inhibits T-cell receptor-driven kinase pathways, T-cell proliferation, and effector functions, ultimately suppressing antimetastatic immunity. The production of TXA_2_ by platelets depends on COX-1, which aspirin can inhibit. As a result, aspirin effectively releases T cells from TXA_2_-mediated suppression. Reproduced from Yang et al^4^ under the terms of the Creative Commons CC BY license.

Cancer metastasis is the leading cause of cancer-related deaths.^67^ Newly metastasized cancer cells are particularly vulnerable to attack from the immune system when they leave the highly immunosuppressive environment typical of established primary tumors. Recently, Yang et al^4^ demonstrated that inhibiting COX-1 activity with aspirin or a selective COX-1 inhibitor enhances immune responses against cancer metastasis in murine models. They discovered that TXA_2_ activates an immunosuppressive pathway in T cells via the guanine nucleotide exchange factor ARHGEF1 (Fig. 7). Conditional removal of ARHGEF1 in T cells boosts T-cell activation, resulting in immune-mediated clearance and a reduction in lung and liver metastases. They also showed that decreasing TXA_2_ availability with aspirin or by selective deletion of COX-1 in the megakaryocyte-platelet lineage lowers the rate of metastasis.^4^ These findings provide a mechanistic explanation for aspirin’s antimetastatic properties and may provide a rationale for developing more effective, immune-based, antimetastatic therapies in the future.^68^

Activated platelets are also thought to have a prometastatic effect by surrounding cancer cells in the bloodstream, forming aggregates that protect them from immune clearance. Activated platelets also help cancer cells to adhere to the endothelium, facilitating their arrest and exit from blood vessels.^69^ Additionally, platelets release factors such as platelet-derived growth factor, transforming growth factor (TGF)-*ß*, and PGE_2_, which facilitate the epithelial-to-mesenchymal transition of cancer cells.^70,71^

Platelets are crucial in immunological processes.^54,72^ They help defend against microbes, recruit and boost innate immune cells, regulate antigen presentation, and enhance adaptive immunity. Fc*γ*RIIa, a low-affinity IgG Fc receptor, is expressed on neutrophils, monocytes, macrophages, and platelets. Fc*γ*RIIa, 1 of 3 immuno-receptor tyrosine-based activation motif. family members found on platelets, signals alongside the collagen receptor glycoprotein (GP)VI and the C-type lectin receptor 2, which follow a different pathway from G protein-coupled receptors for agonists like thrombin, ADP, and TXA_2_.^73,74^ Activation through ITAMs causes platelets to aggregate, degranulate, and become procoagulant, releasing extracellular vesicles.^75^ Fc*γ*RIIa interacts with immune complexes, bacteria,^76^ and serum amyloid P and C-reactive protein,^77^ which are common in inflammatory conditions.^78^ Immune complexes that interact with Fc*γ*RIIa activate platelets.^79^ Mitrugno et al^80^ found that platelet Fc*γ*RIIa mediates platelet-tumor cell interactions, and tumor cells induce platelet secretion. Cancer cell–derived IgG can activate platelets by directly interacting with platelet Fc*γ*RIIa.^81^ The expression of platelet Fc*γ*RIIa is higher in patients with hepatocellular carcinoma (HCC) than in healthy volunteers. These findings suggest that cancer cell–derived IgG may be a key factor in tumor-associated thrombosis and could serve as a diagnostic biomarker and therapeutic target. Using a selective 12-lipoxygenase (LOX) inhibitor (ML355), it was shown that 12-LOX plays an essential role in activating platelets through Fc*γ*RIIa.^82,83^ This finding suggests a possible antitumor effect of 12-LOX inhibitors in preventing cancer cell–derived IgG-dependent platelet activation and cancer-associated thrombosis.

Platelets act as antigen-presenting cells because they express major histocompatibility complex class I proteins on their surface, which can trigger an adaptive immune response.^72^ They have been shown to transfer major histocompatibility complex class I proteins to tumor cells, creating a “phenotype of false pretenses.” This process allows platelets to disrupt self-recognition and non–self-recognition by natural killer cells, ultimately weakening host defense by preventing the production of interferon gamma.^84^ Emerging research indicates that platelets can express PD-L1, which interacts with PD-1 on T cells to suppress immune responses. Patients with non–small cell lung cancer often have high levels of PD-L1 on their platelets,^85^ and therapies blocking PD-L1/PD-1, such as pembrolizumab, have shown effectiveness in patients with metastatic non–small cell lung cancer.^86^ Additionally, certain tumor cells also express high levels of PD-L1, facilitating immune evasion.^87^

TGF-*ß* and lactate are major platelet-derived soluble factors that obliterate CD4+ and CD8+ T-cell functions. Platelets are the dominant source of functional TGF-*ß*, both systemically and in the tumor microenvironment, through the constitutive expression of the TGF-*ß* docking receptor, GPA repetitions predominant (GARP), rather than through TGF-*ß* secretion per se. Platelet-specific deletion of the GARP-encoding gene *Lrrc32* blunted TGF-*ß* activity at the tumor site and potentiated protective immunity against both melanoma and colon cancer.^88^ Thus, platelets constrain T-cell immunity through a GARP-TGF-*ß* axis, suggesting that a combination of immunotherapy with platelet inhibitors could represent a therapeutic strategy against cancer.

Platelets serve as crucial initiators of neutrophil extracellular trap (NET) formation. NETs consist of nuclear DNA expelled during a highly coordinated process, which is distinct from apoptotic and necrotic cell death. NETs play an instrumental role in tumor progression through interactions with immune cells within the tumor microenvironment, thereby collectively influencing its immune profile.^89^ Crosstalk between platelets and neutrophils/NETs can augment or contribute to immune evasion by providing tumor cells with protection. This involves the formation of a physical barrier by platelets and NETs to shield cancer cells from immune assaults, as well as activating immune cells to generate inhibitory signals.

### VI Aspirin and genetic inhibition of platelet activation in experimental in vivo models

Although aspirin is an irreversible inhibitor of COX isozymes, maintaining a virtually complete and prolonged suppression of platelet COX-1 activity with aspirin in mice is challenging due to faster platelet turnover than in humans. This requires using higher and more frequent doses of aspirin to mimic the clinical effects in humans, which may also affect extra-platelet sources of prostanoid production, such as vascular PGI_2_ biosynthesis. An alternative approach is to develop mouse models deficient in either COX-1 or COX-2 to characterize the individual functions of the COX isozymes.^90^ Interestingly, in initial experiments, it was found that COX-1-null mice did not compensate by upregulating COX-2, nor did COX-2-null mice increase COX-1 expression. In COX-1-null mice, although prostanoid levels were reduced by 99% in most tissues and platelet aggregation was impaired, the impact on health was surprisingly minimal, given that COX-1 is constitutively expressed in most tissues. There were some delays in parturition, and notably, COX-1-null mice did not develop gastric ulcers. In contrast, COX-2-null mice had several developmental abnormalities.^90^

The effects of *Ptgs1* (the gene encoding COX-1) and *Ptgs2* (the gene encoding COX-2) deficiency on intestinal tumorigenesis were studied using COX-1 and COX-2 mice crossed with Min (multiple intestinal neoplasia) mice, which possess a chemically induced nonsense mutation in the *Apc* gene (ie, *Apc*^*Min*/+^ mice).^91^ Both COX-1- and COX-2-null-*Apc*^*Min*/+^ mice displayed a 70%–80% reduction in the number of intestinal polyps.^92^ These findings were consistent with experiments using *Apc*^*Δ716*^ knockout mice, a mouse model of human familial adenomatous polyposis (FAP), in which COX-2 deletion was also associated with reduced polyp formation.^93^ The finding that whole-body COX-1 deletion was associated with a reduced number of intestinal adenomas led to the hypothesis that platelet COX-1 downregulation, associated with reduced TXA_2_ biosynthesis, might contribute to the reduction in intestinal tumorigenesis.^25,94,95^ To test this hypothesis, mice with floxed *Ptgs1*, in which exons 6 and 7 were flanked by loxP sites using transcription activator-like effector nucleases as a genome-editing tool, were generated.^62^ These mice were then bred with *Pf4*-Cre transgenic C57BL/6 mice expressing a codon-improved Cre recombinase under the control of the mouse *Pf4* promoter, resulting in Cre recombinase expression in most megakaryocytes.^62^ In these mice, platelet synthesis of TXA_2_ was almost completely suppressed, and systemic biosynthesis of TXA_2_, as assessed by U-TXM, was also significantly reduced. However, COX-2–dependent prostanoid biosynthesis, as reflected by the major urinary metabolites PGE_2_ and PGI_2_, was not significantly affected. This murine model demonstrated the importance of platelet-derived TXA_2_ in experimental intestinal inflammation and fibrosis.^62^ *Apc*^*Min/*+^ mice show enhanced systemic biosynthesis of TXA_2_,^96^ consistent with platelet activation during the early stage of intestinal tumorigenesis. Mice with a selective deletion of COX-1 in platelets were crossed with *Apc*^*Min/*+^ mice^3^ to achieve a selective suppression of platelet COX-1–dependent TXA_2_ biosynthesis. The resultant mice developed fewer intestinal adenomas than control *Apc*^*Min/*+^ mice, and the adenomas expressed less COX-2, which was associated with reduced systemic PGE_2_ biosynthesis, confirming the importance of platelet COX-1 in early tumorigenesis (Fig. 6).^3^

It has also been shown that colon carcinogens, such as azoxymethane (AOM), increase COX-2 expression, leading to elevated PGE_2_ levels in adenocarcinomas.^97^ In rats, this process begins in the normal colonic mucosa just 1 week after AOM exposure, especially on a high-fat corn oil diet.^98^ Repeated injections of AOM efficiently induce colon tumors, and this protocol is recognized as a rodent model of “sporadic” colon tumor development.^99^ In AOM-induced tumors, elevated COX-2 levels have been confirmed, and traditional NSAIDs or selective COX-2 inhibitors reduce carcinogen-induced lesions.^97^ In wild-type mice, COX-2 expression was observed in the stromal areas of AOM-induced colon tumors. In contrast, no tumors were detected in *Ptgs2*^-*/*-^ mice, suggesting that COX-2 plays a causal role in CRC development in this model.

More recently, the effects of aspirin added to drinking water at concentrations of 400 mg/L and 1200 mg/L were investigated in mice given AOM/dextran sodium sulfate and in *Apc*^*Min/*+^ mice.^100^ Aspirin reduced the development of colorectal tumors in both *Apc*^*Min/*+^ mice and those given AOM/dextran sodium sulfate, depending on the presence of intestinal microbes.^100^ It was reported that *Lysinibacillus sphaericus* in the gut degrades aspirin and reduces its ability to prevent tumor formation in mice. Fecal samples from mice fed aspirin were enriched with beneficial bacteria, with reductions in pathogenic bacteria.^100^ However, the impact of the aspirin dosing regimens and administration schedules on prostanoid biosynthesis was not thoroughly evaluated in this study.

Translating these findings to studies of low-dose aspirin use in humans is challenging because of differences in aspirin pharma-cokinetics and pharmacodynamics between mice and humans. Consequently, it remains uncertain whether the phenotypic changes observed in these animal models can be replicated in humans with low-dose aspirin.

Platelets have also been demonstrated to prime CRC cells (HT29) for pulmonary metastasis in immunodeficient murine models by inducing epithelial-to-mesenchymal transition, an effect that is mitigated by the administration of low-dose aspirin. Other antiplatelet agents may produce similar effects, including DG-041 (an EP3 antagonist) and ticagrelor (a P2Y_12_ receptor antagonist).^101^

### VII Effects of other antiplatelet strategies

Both activated platelets and tumors release ADP, which amplifies platelet responses and promotes an immunosuppressive environment.^5^ Tumor-derived ADP activates platelets via the P2Y_12_ receptor, thereby affecting tumor growth.^102^ Inhibiting platelet P2Y_12_ with drugs such as ticagrelor or by genetic deletion reduces ovarian tumor growth in mice, but this effect disappears when thrombopoiesis is restored.^103,104^ Reducing tumor cell ectoapyrase, which scavenges ADP, increases platelet-driven tumor growth, highlighting the role of ADP.^103^ P2Y_12_ knockdown in ovarian cancer does not affect tumor size, whereas in pancreatic cancer, its inhibition reduces proliferation and enhances chemotherapy sensitivity.^105^ Blocking P2Y_12_ may slow tumor growth through antiplatelet effects and direct actions on tumor cells. Additionally, targeting ADP may prevent platelet activation, which facilitates metastasis. Depleting the kinase involved in ADP signaling reduces lung metastasis in mice.^106^

Chronic hepatitis B virus (HBV) infection represents an important risk factor for HCC.^107^ Its pathogenesis involves both viral and host components. Notably, an inadequate CD8+ T-cell response that does not eradicate the virus but perpetuates chronic hepatic inflammation contributes to the development of HCC.^108^ Platelets promote immune-mediated liver injury by aiding virus-specific CD8+ T-cell accumulation in the liver.^109^ In an HBV transgenic mouse model, long-term antiplatelet therapy with aspirin and the P2Y_12_ blocker clopidogrel reduced intrahepatic HBV-specific CD8+ T cells, inflammation, fibrosis, and HCC development, while also increasing survival without significant side effects.^110^ However, this therapy did not affect chemically induced HCC. This suggests that platelets play a key role in immune-mediated liver cancer, and necroinflammatory reactions are a major cause of hepatocellular transformation during chronic hepatitis.

Additional observational studies and/or randomized controlled trials (RCTs) comparing clopidogrel and aspirin are required to properly assess the chemopreventive potential of platelet P2Y_12_ blockers.

Tumor galectin-3 induces platelet activation via GPVI.^111,112^ Revacept is a competitive antagonist of platelet GPVI^113^ that can block the direct interaction of tumor cell galectin-3 with platelet GPVI.^71^ Acting through this mechanism, revacept prevents the overexpression of COX-2 induced by the interaction between cancer cells and platelets.^71^ Relatively small phase 2 clinical trials of revacept have been completed, but none have included cancer-related endpoints. Inhibition of GPVI with the JAQ1 F(ab')2 antibody reduces platelet-tumor cell interactions and metastasis.^114^

Other potential targets for antiplatelet therapy include EP3, TP, GPIIb/IIIa, and PAR-1 receptors.^115^ Oral blockers of platelet TP, GPIIb/IIIa, and PAR-1 have been developed and tested in large phase 3 trials in patients with cerebrovascular disease or acute coronary syndromes.^23,115^ However, the unfavorable balance between the antithrombotic benefits and bleeding risks, compared with aspirin or added to aspirin, has restrained further development in other therapeutic areas.^23,115^

### VIII Inhibition of platelet activation in clinical studies

Once-daily low-dose aspirin (30–100 mg) profoundly suppresses (>97%) platelet COX-1 activity, inhibiting TXA_2_-dependent platelet activation throughout the dosing interval, while leaving other cellular sources of COX-2–dependent prostanoid biosynthesis, such as the vasculature and inflammatory/cancer sites, largely unaffected.^116,117^ Low-dose aspirin has a long-lasting antiplatelet effect (despite its short half-life of ~20 minutes) because it permanently and irreversibly acetylates a serine residue (Ser-529) just below the catalytic pocket of the COX-1 enzyme. Anucleate platelets have limited capacity for de novo protein synthesis, and their lifespan is 7–10 days in humans, with an estimate that only approximately 10%–12% of platelets in the circulation are newly formed and enter the circulation daily.^1,116^ Moreover, indirect evidence suggests that COX-1 in bone marrow megakaryocytes is similarly acetylated by low-dose daily aspirin, ensuring persistent suppression of COX-1–dependent TXA_2_ biosynthesis throughout the 24-hour dosing interval.^1,116,118^

Aspirin can also irreversibly inhibit COX-2 through the acetylation of Ser-516^119^; however, this requires higher doses adminis-tered repeatedly (ie, 650 mg, 4–6 times daily) because nucleated cells can resynthesize new protein within 4–6 hours. It is also noteworthy that COX-2 is less stable than COX-1 in epithelial cells.^120^

Quantitative proteomic assays using liquid chromatography/tandem mass spectrometry enable the assessment of the degree of acetylation of COX-1 and COX-2 in individual cells.^120–122^ In a short-term pharmacodynamic study of 40 apparently healthy subjects undergoing screening for CRC and receiving enteric-coated aspirin (100 mg daily for 7 days) prior to colonoscopy, COX-1 acetylation was evaluated in platelets and in normal colorectal mucosa 7- and 24-hours postdosing.^120^ A significantly lower percentage of acetylated COX-1 was found in the colorectal mucosa (64%) than in blood platelets (75%) at both time points. While platelet COX-1 acetylation was associated with virtually complete suppression of platelet TXB_2_ production, mucosal COX-1 acetylation was only associated with an average 46% and 35% reduction in PGE_2_ and p-S6 levels, respectively.^120^ The exposure of platelets to a higher concentration of intact acetylsalicylic acid in the presystemic circulation^123^ potentially explains the higher level of COX-1 acetylation in platelets than in the colorectal mucosa.^120^

Establishing the appropriate dose of aspirin for CRC prevention is an important and ongoing issue. The question is whether higher doses of aspirin or a twice daily administration of 100 mg will lead to enhanced anticancer effects by inhibiting COX-2, which is associated with tumor growth and progression.^124^ In a study of 34 patients with CRC who received aspirin 100 mg once daily, 300 mg once daily, or 100 mg twice daily over 3 weeks, the 100 mg once daily effectively suppressed TXA_2_-dependent platelet activation, but the effect on COX-2–dependent PGE_2_ was minimal, as reflected by the urinary excretion of its major enzymatic metabolite, PGEM, or PGE_2_ levels in CRC tissue.^125^ Increasing the aspirin dose to 300 mg once daily or administering 100 mg twice daily did not increase the antiplatelet effects, nor did it enhance the acetylation of COX-2 in CRC tissues or significantly inhibit in vivo PGE_2_ biosynthesis. These findings suggest that direct COX-2 inhibition is unlikely to contribute to the anticancer effects of aspirin at this dose range.^125^ Results of ongoing clinical trials will also inform this discussion.

In the Systematic Evaluation of Aspirin and Fish Oil Polyp Prevention (seAFOod) trial, which assessed the effects of aspirin (300 mg daily) and eicosapentaenoic acid (2000 mg daily), alone and in combination, on adenoma risk, urinary biomarkers of prostanoid biosynthesis (U-PGEM and U-TXM) were assessed (*N* = 707).^46^ Significant temporal variations in U-TXM and urinary PGE2 metabolite levels within individuals were found, with aspirin reducing median U-TXM at 6 months by 74%. In the placebo group, a high baseline level of U-TXM (Q2–4) was associated with increased polyp number and risk of developing subsequent polyps.

Additionally, a low on-treatment U-TXM level (Q1) was associated with decreased colorectal polyp number compared with placebo and with higher on-treatment U-TXM levels. The authors conclude that colorectal polyp risk and treatment response prediction using U-TXM are consistent with a role for platelet activation during early colorectal carcinogenesis.^46^

In participants with recently diagnosed cancer in the Add-Aspirin study, aspirin 100 mg once daily reduced U-TXM (median reductions of 77%–82% across all tumor types), with no additional suppression of U-TXM with aspirin 300 mg once daily compared with the lower dose.^45^ This is consistent with the saturability of platelet COX-1 inactivation at 100 mg once daily.^116^ Of note, for trial participants who were randomly allocated to placebo after the run-in phase of aspirin 100 mg once daily for 8 weeks, U-TXM levels returned to baseline levels, demonstrating persistently abnormal platelet activation 4–6 months after initial cancer diagnosis and treatment.^45^

### IX Completed and ongoing clinical trials of aspirin focusing on cancer outcomes

#### A In apparently healthy subjects or those considered at moderate-high risk of vascular disease

The Women’s Health Study randomly assigned 39,876 initially healthy women aged 45 years or older to receive 100 mg aspirin on alternate days or a placebo and then monitored them for 10 years for a first major cardiovascular event (ie, nonfatal myocardial infarction, nonfatal stroke, or death from cardiovascular causes).^126^ There was a nonsignificant 9% reduction in risk with aspirin (relative risk, 0.91; 95% CI, 0.80–1.03; *P* = .13), perhaps reflecting incomplete suppression of platelet COX-1 by the alternate-day regimen. Moreover, during the scheduled follow-up period, there was no effect of alternate-day aspirin on cancer, including total cancer or CRC.^126^ Posttrial observational follow-up of most participants in the Women’s Health Study, which could be susceptible to posttrial biases, revealed a lower incidence of CRC at 17.5 years.^127^ Incidence of CRC was lower in the aspirin group (HR, 0.80; 95% CI, 0.67–0.97; *P* = .021), primarily due to a reduction in proximal colon cancer (HR, 0.73; 95% CI, 0.55–0.95; *P* = .022). The posttrial reduction in CRC was 42% (HR, 0.58; 95% CI, 0.42–0.80; *P* < .001). There was no extended effect on cancer deaths or colorectal polyps.^127^

In this regard, the results of the ASPREE (Aspirin in Reducing Events in the Elderly) trial,^128^ which evaluated aspirin in an older population, are pertinent. This large, randomized study enrolled ~19,000 apparently healthy elderly individuals (median age, 74 years) and assessed whether daily low-dose aspirin could prolong disability-free survival. After a median follow-up of 4.7 years, this trial was terminated due to futility. The primary outcome measure, a composite including all-cause death, dementia, or persistent physical disability, showed no significant difference between those allocated to aspirin or placebo (HR, 1.01; 95% CI, 0.92–1.11; *P* = .79).^128^ The oncological component of this assessment, in particular, attracted attention. Although there was no significant difference between groups for all incident cancers (HR, 1.04; 95% CI, 0.95–1.14), hematological cancers (HR, 0.98; 95% CI, 0.73–1.30), or all solid cancers (HR, 1.05; 95% CI, 0.95–1.15), aspirin was associated with an increased risk of incident cancer that had metastasized (HR, 1.19; 95% CI, 1.00–1.43) or was stage IV at diagnosis (HR, 1.22; 95% CI, 1.02–1.45).^129^ Cancers and their metastases have disordered blood supplies, and bleeding is not an infrequent consequence at first presentation. For example, in a study of cancer symptoms at presentation and stage of disease, 18% of patients with rectal bleeding, 27% of patients with hematuria, and 55% of patients with hemoptysis had stage IV disease at presentation.^130^ Burden or spread of the disease increases the chance of symptomatic presentation and may explain the overall increase in cancers presenting with metastatic disease. Consistent with this, a more recent analysis of the ASPREE cohort, with a median follow-up of 8.6 years, shows a neutral incident cancer risk (HR, 0.98; 95% CI, 0.92–1.05).^131^ Two further relevant trials are ARRIVE^132^ and ASCEND.^52^ The former recruited 12,546 participants considered to be at moderate risk of a major cardiovascular event. After a median follow-up of 60 months, the primary composite outcome of time to cardiovascular death, myocardial infarction, unstable angina, stroke, or transient ischemic attack was seen in 4.3% of those allocated to aspirin 100 mg daily versus 4.5% of those receiving placebo. Overall mortality was 2.6% in both groups, and no data on cancer outcomes were presented in the primary publication.^132^ ASCEND recruited 15,480 participants with diabetes mellitus without apparent cardiovascular disease at trial entry. After a mean follow-up of 7.4 years, serious vascular events were reduced in those receiving aspirin 100 mg daily compared with placebo (rate ratio, 0.88; 95% CI, 0.79–0.97; *P* = .01). However, no significant difference between the aspirin and placebo groups was detected in the incidence of GI tract cancers (2% in both groups) or in all incident cancers (12% in both groups).^52^ These analyses had limited statistical power to detect the hypothesized effects on cancer outcomes, so follow-up is being continued through central registries.^52^

A summary of trials that have been published over the last decade is presented in Table 1.^52,128,129,132–135^

#### B In subjects with sporadic colorectal adenomas

**Fig. 8:**
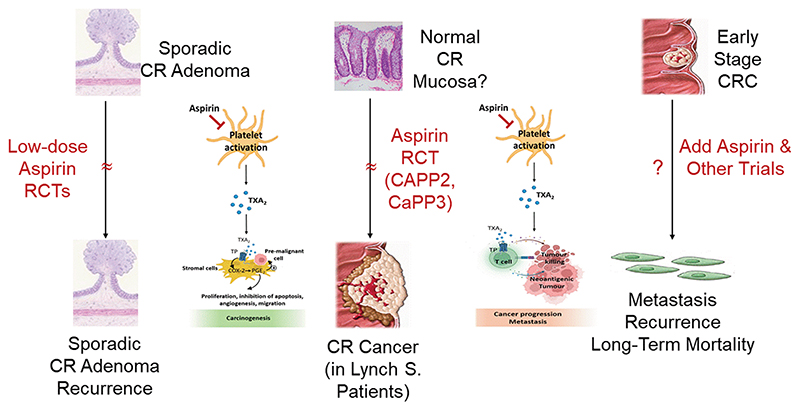
Stages of colorectal carcinogenesis and the chemopreventive effects of aspirin. The figure depicts the 3 clinical settings in which aspirin and other COX inhibitors have been or are being evaluated in randomized clinical trials. The inserts depict the main underlying mechanisms linking platelet activation to CRC development and progression, as discussed in this review. CR, colorectal; Lynch S., Lynch syndrome. Modified from Patrono^141^ with permission from the American Society for Pharmacology and Experimental Therapeutics.

The effect of aspirin on premalignant colorectal lesions (adenomas) has been studied by several groups. A meta-analysis^136^ based on individual participant data from 4 “sporadic” colorectal polyp prevention trials^137–140^ involving ~3000 participants found that aspirin use was associated with a reduction in the adenomatous polyp detection rate of approximately 20% (Fig. 8).^141^ However, it was acknowledged that there was considerable variability across trials in the dose of aspirin, the timing of polyp assessment, and participant eligibility.^136^ Interestingly, low-dose aspirin (81 mg daily) had an antitumor effect similar to that of celecoxib and rofecoxib in preventing sporadic colorectal adenoma recurrence.^137,142,143^ However, the increased risk of cardiovascular side effects associated with COX-2 inhibitors limits their potential long-term use for the primary prevention of cancer,^144,145^ but not necessarily for the treatment of more advanced disease, where the balance of risks and benefits is different.

Three further trials have subsequently been published^146–148^ with variable results, suggesting the need for both additional mechanistic studies and further analyses to fully understand the potential benefits of aspirin at the individual or subgroup level. Emerging themes include the classification of colorectal polyps as either adenomatous or serrated, as data suggest that the effect of aspirin might vary by colorectal polyp type and that colorectal polyp number might be a more relevant measure of chemoprevention efficacy than the previously used adenoma detection rate.^147^

#### C In patients with Lynch syndrome: The concerted action polyp prevention studies

The most convincing trial data relating to aspirin and cancer prevention has resulted from the genetically targeted European concerted action polyp prevention (CAPP) trials; launched in 1993 with an initial focus on FAP was the CAPP1 trial of aspirin (600 mg/d) and/or resistant starch (RS) (30 g/d) for 1–12 years in patients aged 10–21 years, with a 2 × 2 factorial design.^149^ The primary endpoint was polyp number in the rectum and sigmoid colon (at the end of intervention), and the major secondary endpoint was the size of the largest polyp. A total of 206 randomized FAP patients commenced an intervention; 133 had at least 1 follow-up endoscopy and were therefore included in the primary analysis. Neither intervention significantly reduced polyp number: aspirin relative risk = 0.77 (95% CI, 0.54–1.10 vs nonaspirin arms); RS relative risk = 1.05 (95% CI, 0.73–1.49 vs non-RS arms). There was a trend toward a smaller size of the largest polyp in patients treated with aspirin compared with those treated with nonaspirin: mean 3.0 mm versus 6.0 mm, respectively, in patients treated for more than 1 year (*P* = .02).^149^

The discovery in 1993 of the DNA mismatch repair genes underlying hereditary nonpolyposis colon cancer,^150^ later renamed Lynch syndrome, opened the way to a targeted chemoprevention trial with cancer as an endpoint. Lynch syndrome is relatively common, affecting over 1 in 300 people, and is mechanistically a model for the approximately 1 in 6 CRCs with mismatch repair deficiency. The tumors are highly immunogenic due to the generation of frameshift peptides caused by insertions/deletions in repetitive DNA (microsatellites).

CAPP2 used the same interventions of 600 mg of aspirin and/or 30 g of RS versus placebo in a factorial design in Lynch syndrome carriers, treated for 2–4 years with a planned 10-year unblinding. At the end of the intervention period, there was no effect on neoplasia (polyps and cancers combined),^151^ but an interim analysis when the first recruits reached 10 years revealed a significant effect in the per-protocol group.^151^ After a mean follow-up of 4.5 years, once daily aspirin (600 mg) for >2 years reduced CRC incidence by ~60% (HR, 0.41; 95% CI, 0.19–0.86; *P* = .02), with a similar effect across all Lynch syndrome tumors (HR, 0.45; 95% CI, 0.26–0.79; *P* = .005).^151^ The planned 10-year follow-up confirmed a significant reduction in CRC incidence in both intention-to-treat and per-protocol analyses (Fig. 8).^152^ For those who achieved 2 years of therapy, analyses gave an HR of 0.56 (95% CI, 0.34–0.91; *P* = .019) and an incidence rate ratio of 0.50 (95% CI, 0.31–0.82; *P* = .0057), leading the UK National Institute for Health and Care Excellence to recommend “considering daily aspirin, to be taken for >2 years, to prevent CRC in people with Lynch syndrome.”

Consistent with this recommendation, an analysis of 1858 Lynch syndrome gene carriers from the Colon Cancer Family Registry^153^ showed a reduction in CRC incidence among those who self-reported aspirin use (≥5 years of use: HR, 0.25; 95% CI, 0.10–0.62; *P* = .003). Additionally, supporting the platelet hypothesis, a secondary analysis of the CAPP2 data showed an increased risk of CRC with a higher BMI versus normal/low BMI, but only in the placebo group,^154^ suggesting that aspirin abrogated the risk associated with obesity, which is known to enhance platelet activation.^42^

The results of the CAPP3 noninferiority trial comparing aspirin 100 mg daily with aspirin 300 and 600 mg daily in Lynch syndrome carriers have recently been reported. A total of 1866 eligible gene carriers have now been followed for a minimum of 5 years, with preliminary analysis supporting the noninferiority of the 100 mg dose (J. Burn et al, publication under review). After an average of 7 years of follow-up, there were 216 Lynch syndrome cancers, including 91 CRCs, among 176 recruits. The number of cancers in the 100 mg group achieved noninferiority for the predefined threshold when assessed for cancer burden using negative binomial regression (J. Burn et al, publication under review). This finding provides clinical support for the platelet hypothesis and opens the way to wider use of low-dose aspirin in people at increased risk of CRC.

#### D In patients with early-stage solid cancers

The 2009 Antithrombotic Trialists’ Collaboration meta-analysis of randomized trials for vascular prevention was the first to identify a potential benefit of aspirin for nonvascular death (rate ratio, 0.93; 95% CI, 0.85–1.02 in 6 primary prevention trials; rate ratio, 0.85; 95% CI, 0.66–1.08 in 16 secondary prevention trials).^155^ Subsequent meta-analyses of post hoc cancer outcomes in the long-term follow-up of the same aspirin trials showed reductions in cancer incidence, metastasis, and cancer mortality.^156–158^

The reduction in metastasis led to trials evaluating aspirin as an adjuvant treatment following radical therapy. The largest of these is the Add-Aspirin basket trial.^159^ The protocol encompasses 4 individually powered phase 3 trials for early-stage breast, colorectal, gastroesophageal, and prostate cancer. Participants complete radical therapy and, following a run-in phase of aspirin 100 mg daily for 8 weeks (designed to assess feasibility and tolerability), are randomly allocated to aspirin 100 mg, aspirin 300 mg, or placebo daily for 5 years. All the trials have completed recruitment, with over 10,000 participants registered. The primary outcome measure for each trial is disease-free survival (DFS), with a coprimary outcome of overall survival 15 years after randomization across all 4 tumor types, which will assess not only the effects on the original cancer but also the prevention of second cancers and cardiovascular effects.^159^

The majority of the other trials are limited to CRC, with 2 distinct approaches taken. Some trials restricted recruitment to patients with tumors harboring a PIK3CA mutation, as a large cohort study showed that the benefits of aspirin after a diagnosis of CRC were significantly greater in patients with PIK3CA-mutated tumors compared with those with wild-type tumors (cancer-specific survival: HR, 0.18; 95% CI, 0.006–0.61; *P* < .001; overall survival: HR, 0.54; 95% CI, 0.31–0.94; *P* = .01), though the mechanism of action was unclear.^160^ Other trials, including Add-Aspirin, prospectively planned adequately powered, molecularly selected subgroup analyses,^159^ as the PIK3CA data could not be replicated across all datasets.^161^

The first results emerged from the ASCOLT (aspirin after completion of standard adjuvant therapy for colorectal cancer) trial, a molecularly unselected cohort of 1587 participants with Dukes’ C or high-risk Dukes’ B CRC, in which allocation to aspirin (200 mg once daily for 3 years) did not significantly improve DFS (HR, 0.91; 95% CI, 0.73–1.13; *P* = .38).^133^ However, in molecularly selected cohorts, the ALASCCA (adjuvant low dose aspirin in colorectal cancer) trial, in which inclusion was restricted to patients who had either a hotspot mutation in exons 9 and 20 of PIK3CA (group A) or another somatic variant in PIK3CA, PIK3R1, or PTEN (group B), allocation to aspirin (160 mg once daily for 3 years) resulted in a significant reduction in CRC recurrence (primary endpoint) and improved DFS (a secondary endpoint).^135^ The estimated 3-year cumulative incidence of recurrence was 7.7% with aspirin and 14.1% with placebo (HR, 0.49; 95% CI, 0.24–0.98; *P* = .04) among patients with group A alterations, and 7.7% and 16.8%, respectively (HR, 0.42; 95% CI, 0.21–0.83) among those with group B alterations. The estimated 3-year DFS was 88.5% with aspirin and 81.4% with placebo (HR, 0.61; 95% CI, 0.34–1.08) among patients with group A alterations, and 89.1% and 78.7%, respectively (HR, 0.51; 95% CI, 0.29–0.88) among those with group B alterations.^135^ If these promising results are confirmed in other studies, this molecularly selected approach to aspirin use after a CRC diagnosis is likely to become a standard of care where molecular testing is available. A meta-analysis of all adjuvant CRC aspirin trials is planned, which will address both molecular stratification and other factors, including toxicity, likely to impact the benefit:risk profile of aspirin therapy.^162^

A breast cancer trial (Alliance A011502) evaluating aspirin has also been reported, in which over 3000 participants were randomized.^134^ Initially, recruitment was restricted to those within 1 year of diagnosis, but this time frame was extended up to 10 years after diagnosis in some groups to bolster recruitment. The trial was stopped, and participants were taken off treatment after the results from a preplanned interim analysis crossed a predefined futility bound. Invasive DFS events were more frequent in the group allocated to aspirin (HR, 1.27; 95% CI, 0.99–1.63).^134^ The interpretation of this trial requires careful consideration for several reasons: first, as described above, the effect of aspirin on cancer outcomes is most likely mediated through inhibition of platelet activation, with potentially different (and temporally different) downstream effects on primary carcinogenesis and the prevention of metastases. Second, commencing aspirin several years after a cancer diagnosis is not aligned with conventional thinking about adjuvant therapies, which are normally started within weeks of radical treatment with the aim of preventing metastasis formation. Third, aspirin will cause an immediate increase in the risk of severe bleeding, particularly in older individuals, and the wider clinical implications, for example, bleeding from previously undiagnosed malignant lesions, cannot be discounted. Furthermore, given the results of the ALASCCA trial demonstrating the importance of PIK3CA mutations and somatic variants in the phosphatidylinositol 3-kinase (PI3K) pathway in response to aspirin after CRC,^135^ examining PIK3CA mutations in this cohort is of interest.

The extremely variable characteristics of completed RCTs reviewed above are summarized in Table 1.

### X Perspectives

The experimental and clinical evidence we reviewed supports a previously unrecognized role for platelet activation in the early stage of colorectal carcinogenesis, as well as in cancer progression and metastasis.^141,163^ These findings, in turn, support the potential use of low-dose aspirin in cancer prevention and treatment. Although TXA_2_-dependent platelet activation is the most thoroughly investigated mechanism and the established drug target of the antiplatelet effect of low-dose aspirin, it seems biologically plausible that other pathways of platelet activation, such as the ADP-P2Y_12_ pathway, may play a similar, and possibly complementary, role.

The potential association between the use of platelet P2Y_12_ blockers (ie, clopidogrel, prasugrel, and ticagrelor) and the risk of CRC has been scarcely investigated because of the previous lack of a clear rationale, more limited use compared with aspirin, and shorter duration of RCTs. The only observational study that tested the potential anticancer effect of a P2Y_12_ blocker is a nested case-control study using a primary care database in Spain, where it was found that clopidogrel use, alone or combined with low-dose aspirin, lowered the risk of CRC by 20%, a level similar to that seen with low-dose aspirin in similar observational studies.^164^ Although these findings support the hypothesis that platelet in-hibition is an effective strategy for preventing CRC, large RCTs of clopidogrel or ticagrelor have not been performed against a pla-cebo but only against aspirin or on top of aspirin, and none of these trials included cancer as a primary or secondary endpoint.

Exploring the potential chemoprevention effects of other antiplatelet drugs, such as clopidogrel, would offer the prospect of (1) having an alternative to aspirin that is low-cost and generic; (2) potentially enhancing cancer prevention efficacy by combining aspirin and clopidogrel (dual antiplatelet therapy) in selected clinical settings, for example, in the adjuvant setting; and (3) revisiting the balance of benefits and harms of antiplatelet therapy for primary prevention.

There are several unanswered questions that require further work. First, understanding the relationship between aspirin response and perturbations in the PI3K signaling pathway, which will be facilitated by current and emerging trial data, is a priority. Second, although data on the role of U-TXM as a biomarker of cancer risk are promising, they require further validation in larger datasets. Third, the finding that low-dose aspirin acts through the immune system to clear metastases^4^ raises the opportunity of investigating wider integration into the therapeutic armamentarium, particularly in combination with, or possibly as an alternative to, prolonged immune checkpoint inhibitor use.^163^

### XI Conclusions

Despite aspirin being synthesized over 125 years ago, novel effects of this drug continue to be discovered, and its potential utility continues to evolve. Data from RCTs support the use of aspirin for the prevention of Lynch syndrome cancers^152^ (J. Burn et al, publication under review) and in the adjuvant setting for patients with CRC whose tumors harbor PI3K pathway mutations.^135^ Aspirin is unlikely to benefit older, unselected populations or late starters, and may be harmful if initiated late. The cancer results of trials evaluating aspirin in nonmolecularly selected groups have highlighted the need for a better understanding of its anticancer mechanism of action and for a more personalized approach. Experimental findings have underscored the crucial role of platelets in promoting inflammation within the tumor micro-environment.^3,62^ This suggests that targeting platelets could represent a valuable therapeutic strategy for cancer prevention, particularly during the early stages of tumor development. This could be achieved with low-dose aspirin and possibly other antiplatelet agents, like P2Y_12_ blockers. The recent finding that a cancer immunosuppressive pathway is regulated by platelet-derived TXA_2_ provides, for the first time, a clear link between the known pharmacology of low-dose aspirin once daily regimens and an established anticancer mechanism to clear metastases.^4^ Furthermore, identifying platelets as key players in tumor development and progression underscores the importance of ongoing research into novel antiplatelet agents with improved safety profiles.^115^

Concurrently, there needs to be a focus on individual risk rather than “disease” specific indications to develop wider prevention strategies. The field of cardio-oncology continues to grow,^165^ with increasing recognition of shared risk factors and common mechanisms of disease. Using U-TXM or other biomarker(s) to identify who might benefit from aspirin is an attractive way forward. Finally, the results of ongoing adjuvant trials of aspirin for cancer treatment and metastasis prevention may further clarify the role of platelet activation in cancer development and progression and widen the scope of long-term antiplatelet therapy.

## Figures and Tables

**Table 1:** RCTs with cancer outcomes as primary or secondary endpoints

Trial	Population/Intervention	Primary Outcome Measure/Result	Cancer-Related Outcomes, if Not the Primary Outcome
ASPREE NCT01038583 (McNeil et al^128,129^)	Community-dwelling population with no cardiovascular disease, dementia, or physical disability aged ≥ 70 y and Hispanic/Black individuals aged ≥ 65 y in the United States (*N* = 19,114). Aspirin 100 mg once daily vs placebo	Composite of death, dementia, or persistent physical disability. Trial terminated for futility after a median follow-up of 4.7 y. Aspirin 21.5 events per 1000 person-years vs placebo 21.2 (HR, 1.01; 95% CI, 0.92–1.11)	Secondary analysis: no difference between groups for all incident cancers (HR, 1.04; 95% CI, 0.95–1.14). Increase in incident cancers that had metastasized (HR, 1.19; 95% CI, 1.00–1.43) or were later stage at presentation
ASCEND NCT00135226 (ASCEND Study Collaborative Group^52^)	Diabetes but no evident cardiovascular disease (*N* = 15,480). Aspirin 100 mg once daily vs placebo	First serious vascular event, including myocardial infarction, stroke, or transient ischemic attack, or any vascular death excluding intracranial hemorrhage. Aspirin 8.5% vs placebo 9.6% (HR, 0.88; 95% CI, 0.79–0.97)	Mean follow-up was 7.4 y; the incidence of GI tract cancer was 2% in both groups and for all cancers: aspirin 11.6% vs placebo 11.5%
ARRIVE NCT00501059 (Gaziano et al^132^)	Males aged >55 y and females aged >60 y with moderate cardiovascular risk score (*N* = 12,546). Aspirin 100 mg once daily vs placebo	Time to first occurrence of cardiovascular death, myocardial infarction, unstable angina, stroke, or transient ischemic attack.	No cancer-related data reported
		Aspirin 4.29% vs placebo 4.48% (HR, 0.96; 95% CI, 0.81–1.13)	
ASCOLT NCT00565708 (Chia et al^133^)	Adjuvant patients with CRC—no molecular selection (*N* = 1547). Aspirin 200 mg once daily for 3 y vs placebo	DFS^a^ 5-y DFS: aspirin 77% vs 74.8% placebo (HR, 0.91; 95% CI, 0.73–1.13)	
Alliance A011502 NCT02927249 (Chen et al^134^)	Initially adjuvant breast cancer patients, but then extended to include some within 10 y of diagnosis (*N* = 3020). Aspirin 300 mg once daily vs placebo	iDFS^b^ Trial stopped for futility at a median follow-up of 33.8 mo. iDFS events: 141 aspirin vs 112 placebo (HR, 1.27; 95% CI, 0.99–1.63)	
ALASCCA NCT02647099 (Martling et al^135^)	Adjuvant patients with CRC whose tumors had a mutation in the PIK3 pathway (*N* = 626). Group A mutations in exons 9 or 20; group B other somatic variants in *PIK3CA, PIK3R1,* or *PTEN.* Aspirin 160 mg once daily vs placebo	CRC recurrence at 3 y. Group A: aspirin 7.7% vs placebo 14.1% (HR, 0.49; 95% CI, 0.24-0.98). Group B: aspirin 7.7% vs placebo 16.8% (HR, 0.42; 95% CI, 0.21–0.83)	

ALASCCA, Adjuvant Low dose Aspirin in Colorectal Cancer; ALLIANCE, Alliance for Clinical Trials in Oncology (Alliance); ARRIVE, Aspirin to Reduce Risk of Initial Vascular Events; ASCOLT, aspirin after completion of standard adjuvant therapy for colorectal cancer; iDFS, invasive disease–free survival.

a Time from randomization to documented CRC recurrence or death from any cause.

b Time from randomization to the first of any 1 of the following: distant recurrence, locoregional recurrence, ipsilateral or contralateral breast cancer, second primary (not breast) invasive cancer, or death from any cause.

## Data Availability

There are no datasets presented in this paper.

## Significance Statement

We reviewed the experimental and clinical evidence supporting a previously unrecognized role of platelet activation in both the early stage of colorectal carcinogenesis and in cancer progression and metastasis. The findings support the use of low-dose aspirin in cancer prevention and treatment. Data from large randomized clinical trials support the use of aspirin for the prevention of Lynch syndrome cancers and in the adjuvant setting for patients with colorectal cancer whose tumors have a mutation in the phosphatidylinositol 3-kinase pathway genes. Although thromboxane A_2_–dependent platelet activation is the most thoroughly investigated mechanism and the established drug target of the antiplatelet effect of low-dose aspirin, it seems biologically plausible that other pathways of platelet activation, such as the ADP-P2Y_12_ pathway, may play a similar and possibly complementary role.

## Abbreviations

AA: arachidonic acid
AOM: azoxymethane
BMI: body mass index
CAPP: concerted action polyp prevention
COX: cyclo-oxygenase
CRC: colorectal cancer
DFS: disease-free survival
EP: E prostanoid receptor
FAP: familial adenomatous polyposis
GARP: glycoprotein A repetitions predominant
GI: gastrointestinal
GP: glycoprotein
HBV: hepatitis B virus
HCC: hepatocellular carcinoma
HR: hazard ratio
IGT: impaired glucose tolerance
LOX: lip-oxygenase
NET: neutrophil extracellular trap
NSAID: nonsteroidal anti-inflammatory drug
PG: prostaglandin
PGI_2_: prostacyclin
PI3K: phosphatidylinositol 3-kinase
RCT: randomized clinical trial
RS: resistant starch
SVE-R: serious vascular event or revascularization
TGF: transforming growth factor
TP: thromboxane A_2_ receptor
TX: thromboxane
TXM: thromboxane metabolite
U-TXM: urinary thromboxane metabolite.

## References

[1] Patrono C, García Rodríguez LA, Landolfi R, Baigent C (2005). Low-dose aspirin for the prevention of atherothrombosis. N Engl J Med.

[2] Rade JJ, Barton BA, Vasan RS (2022). Association of thromboxane generation with survival in aspirin users and nonusers. J Am Coll Cardiol.

[3] Bruno A, Contursi A, Tacconelli S (2022). The specific deletion of cyclooxygenase-1 in megakaryocytes/platelets reduces intestinal polyposis in Apc^Min/+^ mice. Pharmacol Res.

[4] Yang J, Yamashita-Kanemaru Y, Morris BI (2025). Aspirin prevents metastasis by limiting platelet TXA2 suppression of T cell immunity. Nature.

[5] Tao DL, Tassi Yunga S, Williams CD, McCarty OJT (2021). Aspirin and antiplatelet treatments in cancer. Blood.

[6] Simmons DL, Botting RM, Hla T (2004). Cyclooxygenase isozymes: the biology of prostaglandin synthesis and inhibition. Pharmacol Rev.

[7] Kudo I, Murakami M (2005). Prostaglandin E synthase, a terminal enzyme for prostaglandin E2 biosynthesis. J Biochem Mol Biol.

[8] Joo M, Sadikot RT (2012). PGD synthase and PGD2 in immune resposne. Mediators Inflamm.

[9] Funk CD (2001). Prostaglandins and leukotrienes: advances in eicosanoid biology. Science.

[10] Smyth EM, Grosser T, Wang M, Yu Y, FitzGerald GA (2009). Prostanoids in health and disease. J Lipid Res.

[11] Ricciotti E, FitzGerald GA (2011). Prostaglandins and inflammation. Arterioscler Thromb Vasc Biol.

[12] Nakahata N (2008). Thromboxane A2: physiology/pathophysiology, cellular signal transduction and pharmacology. Pharmacol Ther.

[13] Knezevic I, Borg C, Le Breton GC (1993). Identification of Gq as one of the G-proteins which copurify with human platelet thromboxane A2/prostaglandin H2 receptors. J Biol Chem.

[14] Offermanns S, Laugwitz KL, Spicher K, Schultz G (1994). G proteins of the G12 family are activated via thromboxane A2 and thrombin receptors in human platelets. Proc Natl Acad Sci U S A.

[15] Davì G, Patrono C (2007). Platelet activation and atherothrombosis. N Engl J Med.

[16] Thomas DW, Mannon RB, Mannon PJ (1998). Coagulation defects and altered hemodynamic responses in mice lacking receptors for thromboxane A2. J Clin Invest.

[17] Gawaz M, Vogel S (2013). Platelets in tissue repair: control of apoptosis and interactions with regenerative cells. Blood.

[18] Friedman EA, Ogletree ML, Haddad EV, Boutaud O (2015). Understanding the role of prostaglandin E2 in regulating human platelet activity in health and disease. Thromb Res.

[19] Patrignani P, Patrono C (2015). Cyclooxygenase inhibitors: from pharmacology to clinical read-outs. Biochim Biophys Acta.

[20] Fitzpatrick FA, Soberman R (2001). Regulated formation of eicosanoids. J Clin Invest.

[21] Smith WL, Langenbach R (2001). Why there are two cyclooxygenase isozymes. J Clin Invest.

[22] McAdam BF, Catella-Lawson F, Mardini IA, Kapoor S, Lawson JA, FitzGerald GA (1999). Systemic biosynthesis of prostacyclin by cyclooxygenase (COX)-2: the human pharmacology of a selective inhibitor of COX-2. Proc Natl Acad Sci U S A.

[23] Patrono C (2024). Low-dose aspirin for the prevention of atherosclerotic cardiovascular disease. Eur Heart J.

[24] FitzGerald GA, Patrono C (2001). The coxibs, selective inhibitors of cyclooxygenase-2. N Engl J Med.

[25] Patrono C, Patrignani P, Rodríguez LAG (2001). Cyclooxygenase-selective inhibition of prostanoid formation: transducing biochemical selectivity into clinical read-outs. J Clin Invest.

[26] Laine L, Maller ES, Yu C, Quan H, Simon T (2004). Ulcer formation with low-dose enteric-coated aspirin and the effect of COX-2 selective inhibition: a double-blind trial. Gastroenterology.

[27] Bhala N, Emberson J, Merhi A, Coxib and traditional NSAID Trialists’ (CNT) Collaboration (2013). Vascular and upper gastrointestinal effects of non-steroidal anti-inflammatory drugs: meta-analyses of individual participant data from randomised trials. Lancet.

[28] Born GV (1962). Aggregation of blood platelets by adenosine diphosphate and its reversal. Nature.

[29] Born GV, Patrono C (2006). Antiplatelet drugs. Br J Pharmacol.

[30] Patrono C, Ciabattoni G, Pinca E (1980). Low dose aspirin and inhibition of thromboxane B2 production in healthy subjects. Thromb Res.

[31] Patrono C, Ciabattoni G, Pugliese F, Pierucci A, Blair IA, FitzGerald GA (1986). Estimated rate of thromboxane secretion into the circulation of normal humans. J Clin Invest.

[32] FitzGerald GA, Pedersen AK, Patrono C (1983). Analysis of prostacyclin and thromboxane biosynthesis in cardiovascular disease. Circulation.

[33] Patrignani P, Morton H, Cirino M (1989). Fractional conversion of thromboxane A_2_ and B_2_ to urinary 2,3-dinor-thromboxane B_2_ and 11-dehydro-thromboxane B_2_ in the cynomolgus monkey. Biochim Biophys Acta.

[34] Roberts LJ, Sweetman BJ, Oates JA (1981). Metabolism of thromboxane B_2_ in man. Identification of twenty urinary metabolites. J Biol Chem.

[35] Patrono C, Rocca B (2019). Measurement of thromboxane biosynthesis in health and disease. Front Pharmacol.

[36] Ciabattoni G, Pugliese F, Davi G, Pierucci A, Simonetti BM, Patrono C (1989). Fractional conversion of thromboxane B_2_ to urinary 11-dehydrothromboxane B2 in man. Biochim Biophys Acta.

[37] Davì G, Catalano I, Averna M (1990). Thromboxane biosynthesis and platelet function in type II diabetes mellitus. N Engl J Med.

[38] Santilli F, Rocca B, De Cristofaro R (2009). Platelet cyclooxygenase inhibition by low-dose aspirin is not reflected consistently by platelet function assays: implications for aspirin “resistance”. J Am Coll Cardiol.

[39] Roweth HG, Battinelli EM (2021). Lessons to learn from tumor-educated platelets. Blood.

[40] In ‘t Veld SGJG, Arkani M, Post E (2022). Detection and localization of early- and late-stage cancers using platelet RNA. Cancer Cell.

[41] Davì G, Averna M, Catalano I (1992). Increased thromboxane biosynthesis in type IIa hypercholesterolemia. Circulation.

[42] Davì G, Guagnano MT, Ciabattoni G (2002). Platelet activation in obese women: role of inflammation and oxidant stress. JAMA.

[43] Minuz P, Patrignani P, Gaino S (2002). Increased oxidative stress and platelet activation in patients with hypertension and renovascular disease. Circulation.

[44] Nowak J, Murray JJ, Oates JA, FitzGerald GA (1987). Biochemical evidence of a chronic abnormality in platelet and vascular function in healthy individuals who smoke cigarettes. Circulation.

[45] Joharatnam-Hogan N, Hatem D, Cafferty FH (2023). Thromboxane biosynthesis in cancer patients and its inhibition by aspirin: a sub-study of the Add-Aspirin trial. Br J Cancer.

[46] Sun G, Fuller H, Fenton H (2024). The effect of aspirin and eicosapentaenoic acid on urinary biomarkers of prostaglandin E2 synthesis and platelet activation in participants of the seAFOod polyp prevention trial. Int J Cancer.

[47] Morrow JD (2005). Quantification of isoprostanes as indices of oxidant stress and the risk of atherosclerosis in humans. Arterioscler Thromb Vasc Biol.

[48] Morrow JD, Hill KE, Burk RF, Nammour TM, Badr KF, Roberts LJ (1990). A series of prostaglandin F2-like compounds are produced in vivo in humans by a non-cyclooxygenase, free radical-catalyzed mechanism. Proc Natl Acad Sci U S A.

[49] Praticò D, Smyth EM, Violi F, FitzGerald GA (1996). Local amplification of platelet function by 8-Epi prostaglandin F2*α* is not mediated by thromboxane receptor isoforms. J Biol Chem.

[50] Santilli F, Zaccardi F, Liani R (2020). In vivo thromboxane-dependent platelet activation is persistently enhanced in subjects with impaired glucose tolerance. Diabetes Metab Res Rev.

[51] Petrucci G, Buck GA, Rocca B (2024). Thromboxane biosynthesis and future events in diabetes: the ASCEND trial. Eur Heart J.

[52] Bowman L, Mafham M, Wallendszus K, ASCEND Study Collaborative Group (2018). Effects of aspirin for primary prevention in persons with diabetes mellitus. N Engl J Med.

[53] Kiely M, Milne GL, Minas TZ (2022). Urinary thromboxane B2 and lethal prostate cancer in African American men. J Natl Cancer Inst.

[54] Contursi A, Tacconelli S, Di Berardino S, De Michele A, Patrignani P (2024). Platelets as crucial players in the dynamic interplay of inflammation, immunity, and cancer: unveiling new strategies for cancer prevention. Front Pharmacol.

[55] De La Cruz A, Hargrave A, Magadi S (2021). Platelet and erythrocyte extravasation across inflamed corneal venules depend on CD18, neutrophils, and mast cell degranulation. Int J Mol Sci.

[56] Pitchford SC, Yano H, Lever R (2003). Platelets are essential for leukocyte recruitment in allergic inflammation. J Allergy Clin Immunol.

[57] Pitchford SC, Momi S, Giannini S (2005). Platelet P-selectin is required for pulmonary eosinophil and lymphocyte recruitment in a murine model of allergic inflammation. Blood.

[58] Pitchford SC, Momi S, Baglioni S (2008). Allergen induces the migration of platelets to lung tissue in allergic asthma. Am J Respir Crit Care Med.

[59] D’Agostino I, Tacconelli S, Bruno A (2021). Low-dose Aspirin prevents hypertension and cardiac fibrosis when thromboxane A_2_ is unrestrained. Pharmacol Res.

[60] Danese S, de la Motte Cd C, Fiocchi C (2004). Platelets in inflammatory bowel disease: clinical, pathogenic, and therapeutic implications. Am J Gastroenterol.

[61] Petito E, Momi S, Gresele P, Gresele P, Kleiman NS, Lopez JA, Page CP (2017). Platelets in Thrombotic and Non-Thrombotic Disorders.

[62] Sacco A, Bruno A, Contursi A (2019). Platelet-specific deletion of cyclooxygenase-1 ameliorates dextran sulfate sodium-induced colitis in mice. J Pharmacol Exp Ther.

[63] Faour WH, He Y, He QW (2001). Prostaglandin E(2) regulates the level and stability of cyclooxygenase-2 mRNA through activation of p38 mitogen-activated protein kinase in interleukin-1 *β*-treated human synovial fibroblasts. J Biol Chem.

[64] Wang D, DuBois RN (2013). An inflammatory mediator, prostaglandin E2, in colorectal cancer. Cancer J.

[65] Nakanishi M, Rosenberg DW (2013). Multifaceted roles of PGE2 in inflammation and cancer. Semin Immunopathol.

[66] Tessner TG, Muhale F, Riehl TE, Anant S, Stenson WF (2004). Prostaglandin E2 reduces radiation-induced epithelial apoptosis through a mechanism involving AKT activation and bax translocation. J Clin Invest.

[67] Massagué J, Obenauf AC (2016). Metastatic colonization by circulating tumour cells. Nature.

[68] Liuzzo G, Patrono C (2025). Weekly Journal Scan: platelet thromboxane suppression as the mechanism of the anti-metastatic effect of aspirin. Eur Heart J.

[69] Gay LJ, Felding-Habermann B (2011). Contribution of platelets to tumour metastasis. Nat Rev Cancer.

[70] Labelle M, Begum S, Hynes RO (2011). Direct signaling between platelets and cancer cells induces an epithelial-mesenchymal-like transition and promotes metastasis. Cancer Cell.

[71] Dovizio M, Maier TJ, Alberti S (2013). Pharmacological inhibition of platelet-tumor cell cross-talk prevents platelet-induced overexpression of cyclooxygenase-2 in HT29 human colon carcinoma cells. Mol Pharmacol.

[72] Ali RA, Wuescher LM, Worth RG (2015). Platelets: essential components of the immune system. Curr Trends Immunol.

[73] Boylan B, Gao C, Rathore V, Gill JC, Newman DK, Newman PJ (2008). Identification of FcγRIIA as the ITAM-bearing receptor mediating *α*IIb*β*3 outside-in integrin signaling in human platelets. Blood.

[74] Boulaftali Y, Hess PR, Kahn ML, Bergmeier W (2014). Platelet immunoreceptor tyrosine-based activation motif (ITAM) signaling and vascular integrity. Circ Res.

[75] Puhm F, Boilard E, Machlus KR (2021). Platelet extracellular vesicles: beyond the blood. Arterioscler Thromb Vasc Biol.

[76] Arman M, Krauel K, Tilley DO (2014). Amplification of bacteria-induced platelet activation is triggered by FcγRIIA, integrin *α*IIb*β*3, and platelet factor 4. Blood.

[77] Lu J, Marnell LL, Marjon KD, Mold C, Du Clos TW, Sun PD (2008). Structural recognition and functional activation of FcgammaR by innate pentraxins. Nature.

[78] Cloutier N, Allaeys I, Marcoux G (2018). Platelets release pathogenic serotonin and return to circulation after immune complex-mediated sequestration. Proc Natl Acad Sci U S A.

[79] Arman M, Krauel K (2015). Human platelet IgG Fc receptor FcγRIIA in immunity and thrombosis. J Thromb Haemost.

[80] Mitrugno A, Williams D, Kerrigan SW, Moran N (2014). A novel and essential role for FcγRIIa in cancer cell-induced platelet activation. Blood.

[81] Miao S, Shu D, Zhu Y (2019). Cancer cell-derived immunoglobulin G activates platelets by binding to platelet FcγRIIa. Cell Death Dis.

[82] Yeung J, Tourdot BE, Fernandez-Perez P (2014). Platelet 12-LOX is essential for FcγRIIa-mediated platelet activation. Blood.

[83] Luci DK, Jameson JB, Yasgar A (2014). Synthesis and structure-activity relationship studies of 4-((2-hydroxy-3-methoxybenzyl)amino)benzenesulfonamide derivatives as potent and selective inhibitors of 12-lipoxygenase. J Med Chem.

[84] Placke T, Örgel M, Schaller M (2012). Platelet-derived MHC class I confers a pseudonormal phenotype to cancer cells that subverts the antitumor reactivity of natural killer immune cells. Cancer Res.

[85] Hinterleitner C, Strähle J, Malenke E (2021). Platelet PD-L1 reflects collective intratumoral PD-L1 expression and predicts immunotherapy response in non-small cell lung cancer. Nat Commun.

[86] Dang TO, Ogunniyi A, Barbee MS, Drilon A (2016). Pembrolizumab for the treatment of PD-L1 positive advanced or metastatic non-small cell lung cancer. Expert Rev Anticancer Ther.

[87] Juneja VR, McGuire KA, Manguso RT (2017). PD-L1 on tumor cells is sufficient for immune evasion in immunogenic tumors and inhibits CD8 T cell cytotoxicity. J Exp Med.

[88] Rachidi S, Metelli A, Riesenberg B (2017). Platelets subvert T cell immunity against cancer via GARP-TGFβ axis. Sci Immunol.

[89] Li N, Yin C, Tao J (2025). Neutrophil extracellular traps in tumor metastasis: mechanisms, and therapeutic implications. Discov Oncol.

[90] Langenbach R, Loftin C, Lee C, Tiano H (1999). Cyclooxygenase knockout mice: models for elucidating isoform-specific functions. Biochem Pharmacol.

[91] Moser AR, Luongo C, Gould KA, McNeley MK, Shoemaker AR, Dove WF (1995). ApcMin: a mouse model for intestinal and mammary tumorigenesis. Eur J Cancer.

[92] Chulada PC, Thompson MB, Mahler JF (2000). Genetic disruption of Ptgs-1, as well as Ptgs-2, reduces intestinal tumorigenesis in Min mice. Cancer Res.

[93] Oshima M, Dinchuk JE, Kargman SL (1996). Suppression of intestinal polyposis in Apc delta716 knockout mice by inhibition of cyclooxygenase 2 (COX-2). Cell.

[94] Thun MJ, Henley SJ, Patrono C (2002). Nonsteroidal anti-inflammatory drugs as anticancer agents: mechanistic, pharmacologic, and clinical issues. J Natl Cancer Inst.

[95] Patrignani P, Patrono C (2016). Aspirin and cancer. J Am Coll Cardiol.

[96] Dovizio M, Tacconelli S, Ricciotti E (2012). Effects of celecoxib on prostanoid biosynthesis and circulating angiogenesis proteins in familial adenomatous polyposis. J Pharmacol Exp Ther.

[97] Rosenberg DW, Giardina C, Tanaka T (2009). Mouse models for the study of colon carcinogenesis. Carcinogenesis.

[98] Singh J, Hamid R, Reddy BS (1997). Dietary fat and colon cancer: modulation of cyclooxygenase-2 by types and amount of dietary fat during the post-initiation stage of colon carcinogenesis. Cancer Res.

[99] Neufert C, Becker C, Neurath MF (2007). An inducible mouse model of colon carcinogenesis for the analysis of sporadic and inflammation-driven tumor progression. Nat Protoc.

[100] Zhao R, Coker OO, Wu J (2020). Aspirin reduces colorectal tumor development in mice and gut microbes reduce its bioavailability and chemopreventive effects. Gastroenterology.

[101] Guillem-Llobat P, Dovizio M, Bruno A (2016). Aspirin prevents colorectal cancer metastasis in mice by splitting the crosstalk between platelets and tumor cells. Oncotarget.

[102] Xi Y, Min Z, Liu M, Lin X, Yuan ZH (2025). Role and recent progress of P2Y12 receptor in cancer development. Purinergic Signal.

[103] Cho MS, Bottsford-Miller J, Vasquez HG (2012). Platelets increase the proliferation of ovarian cancer cells. Blood.

[104] Mitrugno A, McCarty OJT (2017). Ticagrelor breaks up the tumor-platelet party. Blood.

[105] Elaskalani O, Domenichini A, Abdol Razak NB, Dye DE, Falasca M, Metharom P (2020). Antiplatelet drug ticagrelor enhances chemotherapeutic efficacy by targeting the novel P2Y12-AKT pathway in pancreatic cancer cells. Cancers (Basel).

[106] Kamiyama M, Shirai T, Tamura S (2017). ASK1 facilitates tumor metastasis through phosphorylation of an ADP receptor P2Y12 in platelets. Cell Death Differ.

[107] Akinyemiju T, Abera S, Ahmed M, Global Burden of Disease Liver Cancer Collaboration (2017). The burden of primary liver cancer and underlying etiologies from 1990 to 2015 at the global, regional, and national level: results from the Global Burden of Disease Study 2015. JAMA Oncol.

[108] Neumann-Haefelin C, Spangenberg HC, Blum HE, Thimme R (2007). Host and viral factors contributing to CD8+ T cell failure in hepatitis C virus infection. World J Gastroenterol.

[109] Aiolfi R, Sitia G (2015). Chronic hepatitis B: role of anti-platelet therapy in inflammation control. Cell Mol Immunol.

[110] Sitia G, Aiolfi R, Di Lucia P (2012). Antiplatelet therapy prevents hepatocellular carcinoma and improves survival in a mouse model of chronic hepatitis B. Proc Natl Acad Sci U S A.

[111] Nicolson PLR, Nock SH, Hinds J (2021). Low-dose Btk inhibitors selectively block platelet activation by CLEC-2. Haematologica.

[112] Rigg RA, Aslan JE, Healy LD (2016). Oral administration of Bruton’s tyrosine kinase inhibitors impairs GPVI-mediated platelet function. Am J Physiol Cell Physiol.

[113] Ungerer M, Rosport K, Bültmann A (2011). Novel antiplatelet drug revacept (Dimeric Glycoprotein VI-Fc) specifically and efficiently inhibited collagen-induced platelet aggregation without affecting general hemostasis in humans. Circulation.

[114] Mammadova-Bach E, Gil-Pulido J, Sarukhanyan E (2020). Platelet glycoprotein VI promotes metastasis through interaction with cancer cell-derived galectin-3. Blood.

[115] Gawaz M, Geisler T, Borst O (2023). Current concepts and novel targets for anti-platelet therapy. Nat Rev Cardiol.

[116] Patrignani P, Filabozzi P, Patrono C (1982). Selective cumulative inhibition of platelet thromboxane production by low-dose aspirin in healthy subjects. J Clin Invest.

[117] Capone ML, Tacconelli S, Sciulli MG (2004). Clinical pharmacology of platelet, monocyte, and vascular cyclooxygenase inhibition by naproxen and low-dose aspirin in healthy subjects. Circulation.

[118] Giaretta A, Rocca B, Di Camillo B, Toffolo GM, Patrono C (2017). In silico modeling of the antiplatelet pharmacodynamics of low-dose aspirin in health and disease. Clin Pharmacol Ther.

[119] Lecomte M, Laneuville O, Ji C, DeWitt DL, Smith WL (1994). Acetylation of human prostaglandin endoperoxide synthase-2 (cyclooxygenase-2) by aspirin. J Biol Chem.

[120] Patrignani P, Sacco A, Sostres C (2017). Low-dose aspirin acetylates cyclooxygenase-1 in human colorectal mucosa: implications for the chemoprevention of colorectal cancer. Clin Pharmacol Ther.

[121] Patrignani P, Tacconelli S, Piazuelo E (2014). Reappraisal of the clinical pharmacology of low-dose aspirin by comparing novel direct and traditional indirect biomarkers of drug action. J Thromb Haemost.

[122] Tacconelli S, Contursi A, Falcone L (2020). Characterization of cyclooxygenase-2 acetylation and prostanoid inhibition by aspirin in cellular systems. Biochem Pharmacol.

[123] Pedersen AK, Fitzgerald GA (1984). Dose-related kinetics of aspirin. Presystemic acetylation of platelet cyclooxygenase. N Engl J Med.

[124] Wang D, DuBois RN (2010). The role of COX-2 in intestinal inflammation and colorectal cancer. Oncogene.

[125] Patrignani P, Tacconelli S, Contursi A (2024). Optimizing aspirin dose for colorectal cancer patients through deep phenotyping using novel biomarkers of drug action. Front Pharmacol.

[126] Ridker PM, Cook NR, Lee IM (2005). A randomized trial of low-dose aspirin in the primary prevention of cardiovascular disease in women. N Engl J Med.

[127] Cook NR, Lee IM, Zhang SM, Moorthy MV, Buring JE (2013). Alternate-day, low-dose aspirin and cancer risk: long-term observational follow-up of a randomized trial. Ann Intern Med.

[128] McNeil JJ, Woods RL, Nelson MR (2018). Effect of aspirin on disability-free survival in the healthy elderly. N Engl J Med.

[129] McNeil JJ, Gibbs P, Orchard SG (2021). Effect of aspirin on cancer incidence and mortality in older adults. J Natl Cancer Inst.

[130] Koo MM, Swann R, McPhail S (2020). Presenting symptoms of cancer and stage at diagnosis: evidence from a cross-sectional, population-based study. Lancet Oncol.

[131] Orchard S Low-dose aspirin for primary prevention of cancer in older adults: ASPREE after 8 years.

[132] Gaziano JM, Brotons C, Coppolecchia R (2018). Use of aspirin to reduce risk of initial vascular events in patients at moderate risk of cardiovascular disease (ARRIVE): a randomised, double-blind, placebo-controlled trial. Lancet.

[133] Chia JWK, Segelov E, Deng Y (2025). Aspirin after completion of standard adjuvant therapy for colorectal cancer (ASCOLT): an international, multi-centre, phase 3, randomised, double-blind, placebo-controlled trial. Lancet Gastroenterol Hepatol.

[134] Chen WY, Ballman KV, Partridge AH (2024). Aspirin vs placebo as adjuvant therapy for breast cancer: the Alliance A011502 randomized trial. JAMA.

[135] Martling A, Hed Myrberg I, Nilbert M, ALASCCA Study Group (2025). Low-dose aspirin for PI3K-altered localized colorectal cancer. N Engl J Med.

[136] Cole BF, Logan RF, Halabi S (2009). Aspirin for the chemoprevention of colorectal adenomas: meta-analysis of the randomized trials. J Natl Cancer Inst.

[137] Baron JA, Cole BF, Sandler RS (2003). A randomized trial of aspirin to prevent colorectal adenomas. N Engl J Med.

[138] Cole BF, Baron JA, Sandler RS, Polyp Prevention Study Group (2007). Folic acid for the prevention of colorectal adenomas: a randomized clinical trial. JAMA.

[139] Sandler RS, Halabi S, Baron JA (2003). A randomized trial of aspirin to prevent colorectal adenomas in patients with previous colorectal cancer. N Engl J Med.

[140] Benamouzig R, Deyra J, Martin A (2003). Daily soluble aspirin and prevention of colorectal adenoma recurrence: one-year results of the APACC trial. Gastroenterology.

[141] Patrono C (2023). Cyclooxygenase inhibitors and cancer: the missing pieces. J Pharmacol Exp Ther.

[142] Bertagnolli MM, Eagle CJ, Zauber AG (2006). Celecoxib for the prevention of sporadic colorectal adenomas. N Engl J Med.

[143] Baron JA, Sandler RS, Bresalier RS (2006). A randomized trial of rofecoxib for the chemoprevention of colorectal adenomas. Gastroenterology.

[144] Grosser T, Fries S, FitzGerald GA (2006). Biological basis for the cardiovascular consequences of COX-2 inhibition: therapeutic challenges and opportunities. J Clin Invest.

[145] Kearney PM, Baigent C, Godwin J, Halls H, Emberson JR, Patrono C (2006). Do selective cyclo-oxygenase-2 inhibitors and traditional non-steroidal anti-inflammatory drugs increase the risk of atherothrombosis? Meta-analysis of randomised trials. BMJ.

[146] Ishikawa H, Mutoh M, Suzuki S (2014). The preventive effects of low-dose enteric-coated aspirin tablets on the development of colorectal tumours in Asian patients: a randomised trial. Gut.

[147] Hull MA, Sprange K, Hepburn T (2018). Eicosapentaenoic acid and aspirin, alone and in combination, for the prevention of colorectal adenomas (seA-FOod Polyp Prevention trial): a multicentre, randomised, double-blind, placebo-controlled, 2 × 2 factorial trial. Lancet.

[148] Pommergaard HC, Burcharth J, Rosenberg J, Raskov H (2016). Aspirin, calcitriol, and calcium do not prevent adenoma recurrence in a randomized controlled trial. Gastroenterology.

[149] Burn J, Gerdes AM, Macrae F (2011). Long-term effect of aspirin on cancer risk in carriers of hereditary colorectal cancer: an analysis from the CAPP2 randomised controlled trial. Lancet.

[150] Fishel R, Lescoe MK, Rao MR (1993). The human mutator gene homolog MSH2 and its association with hereditary nonpolyposis colon cancer. Cell.

[151] Burn J, Bishop DT, Mecklin JP (2008). Effect of aspirin or resistant starch on colorectal neoplasia in the Lynch syndrome. N Engl J Med.

[152] Burn J, Sheth H, Elliott F (2020). Cancer prevention with aspirin in hereditary colorectal cancer (Lynch syndrome), 10-year follow-up and registry-based 20-year data in the CAPP2 study: a double-blind, randomised, placebo-controlled trial. Lancet.

[153] Ait Ouakrim D, Dashti SG, Chau R (2015). Aspirin, ibuprofen, and the risk of colorectal cancer in Lynch syndrome. J Natl Cancer Inst.

[154] Movahedi M, Bishop DT, Macrae F (2015). Obesity, aspirin, and risk of colorectal cancer in carriers of hereditary colorectal cancer: a prospective investigation in the CAPP2 study. J Clin Oncol.

[155] Baigent C, Blackwell L, Collins R, Antithrombotic Trialists’ (ATT) Collaboration (2009). Aspirin in the primary and secondary prevention of vascular disease: collaborative meta-analysis of individual participant data from randomised trials. Lancet.

[156] Algra AM, Rothwell PM (2012). Effects of regular aspirin on long-term cancer incidence and metastasis: a systematic comparison of evidence from observational studies versus randomised trials. Lancet Oncol.

[157] Rothwell PM, Fowkes FG, Belch JF, Ogawa H, Warlow CP, Meade TW (2011). Effect of daily aspirin on long-term risk of death due to cancer: analysis of individual patient data from randomised trials. Lancet.

[158] Rothwell PM, Wilson M, Price JF, Belch JF, Meade TW, Mehta Z (2012). Effect of daily aspirin on risk of cancer metastasis: a study of incident cancers during randomised controlled trials. Lancet.

[159] Coyle C, Cafferty FH, Rowley S, Add-Aspirin investigators (2016). ADD-ASPIRIN: a phase III, double-blind, placebo controlled, randomised trial assessing the effects of aspirin on disease recurrence and survival after primary therapy in common non-metastatic solid tumours. Contemp Clin Trials.

[160] Liao X, Lochhead P, Nishihara R (2012). Aspirin use, tumor PIK3CA mutation, and colorectal cancer survival. N Eng J Med.

[161] Paleari L, Puntoni M, Clavarezza M, DeCensi M, Cuzick J, DeCensi A (2016). PIK3CA mutation, aspirin use after diagnosis and survival of colorectal cancer. a systematic review and meta-analysis of epidemiological studies. Clin Oncol (R Coll Radiol).

[162] Burdett S, Fisher D, Tierney J, Meade A, Nankivell M, Langley R Aspirin after radical therapy for colorectal cancer: a prospective meta-analysis.

[163] Langley RE, Burn J (2025). Understanding how aspirin prevents metastasis. N Engl J Med.

[164] Rodríguez-Miguel A, García-Rodríguez LA, Gil M, Montoya H, Rodríguez-Martín S, de Abajo FJ (2019). Clopidogrel and low-dose aspirin, alone or together, reduce risk of colorectal cancer. Clin Gastroenterol Hepatol.

[165] Camilli M, Maggio L, Tinti L (2025). Cardio-oncology: emerging concepts in cardiovascular sequelae of cancer therapies, translational research and reverse cardio-oncology. Eur Cardiol.

